# Search for lepton flavour violating decays of heavy resonances and quantum black holes to an $\mathrm {e}\mu$ pair in proton–proton collisions at $\sqrt{s}=8~\text {TeV}$

**DOI:** 10.1140/epjc/s10052-016-4149-y

**Published:** 2016-06-10

**Authors:** V. Khachatryan, A. M. Sirunyan, A. Tumasyan, W. Adam, E. Asilar, T. Bergauer, J. Brandstetter, E. Brondolin, M. Dragicevic, J. Erö, M. Flechl, M. Friedl, R. Frühwirth, V. M. Ghete, C. Hartl, N. Hörmann, J. Hrubec, M. Jeitler, V. Knünz, A. König, M. Krammer, I. Krätschmer, D. Liko, T. Matsushita, I. Mikulec, D. Rabady, N. Rad, B. Rahbaran, H. Rohringer, J. Schieck, R. Schöfbeck, J. Strauss, W. Treberer-Treberspurg, W. Waltenberger, C.-E. Wulz, V. Mossolov, N. Shumeiko, J. Suarez Gonzalez, S. Alderweireldt, T. Cornelis, E. A. De Wolf, X. Janssen, A. Knutsson, J. Lauwers, S. Luyckx, M. Van De Klundert, H. Van Haevermaet, P. Van Mechelen, N. Van Remortel, A. Van Spilbeeck, S. Abu Zeid, F. Blekman, J. D’Hondt, N. Daci, I. De Bruyn, K. Deroover, N. Heracleous, J. Keaveney, S. Lowette, L. Moreels, A. Olbrechts, Q. Python, D. Strom, S. Tavernier, W. Van Doninck, P. Van Mulders, G. P. Van Onsem, I. Van Parijs, P. Barria, H. Brun, C. Caillol, B. Clerbaux, G. De Lentdecker, W. Fang, G. Fasanella, L. Favart, R. Goldouzian, A. Grebenyuk, G. Karapostoli, T. Lenzi, A. Léonard, T. Maerschalk, A. Marinov, L. Perniè, A. Randle-conde, T. Seva, C. Vander Velde, P. Vanlaer, R. Yonamine, F. Zenoni, F. Zhang, K. Beernaert, L. Benucci, A. Cimmino, S. Crucy, D. Dobur, A. Fagot, G. Garcia, M. Gul, J. Mccartin, A. A. Ocampo Rios, D. Poyraz, D. Ryckbosch, S. Salva, M. Sigamani, M. Tytgat, W. Van Driessche, E. Yazgan, N. Zaganidis, S. Basegmez, C. Beluffi, O. Bondu, S. Brochet, G. Bruno, A. Caudron, L. Ceard, C. Delaere, D. Favart, L. Forthomme, A. Giammanco, A. Jafari, P. Jez, M. Komm, V. Lemaitre, A. Mertens, M. Musich, C. Nuttens, L. Perrini, K. Piotrzkowski, A. Popov, L. Quertenmont, M. Selvaggi, M. Vidal Marono, N. Beliy, G. H. Hammad, W. L. Aldá Júnior, F. L. Alves, G. A. Alves, L. Brito, M. Correa Martins Junior, M. Hamer, C. Hensel, A. Moraes, M. E. Pol, P. Rebello Teles, E. Belchior Batista Das Chagas, W. Carvalho, J. Chinellato, A. Custódio, E. M. Da Costa, D. De Jesus Damiao, C. De Oliveira Martins, S. Fonseca De Souza, L. M. Huertas Guativa, H. Malbouisson, D. Matos Figueiredo, C. Mora Herrera, L. Mundim, H. Nogima, W. L. Prado Da Silva, A. Santoro, A. Sznajder, E. J. Tonelli Manganote, A. Vilela Pereira, S. Ahuja, C. A. Bernardes, A. De Souza Santos, S. Dogra, T. R. Fernandez Perez Tomei, E. M. Gregores, P. G. Mercadante, C. S. Moon, S. F. Novaes, Sandra S. Padula, D. Romero Abad, J. C. Ruiz Vargas, A. Aleksandrov, R. Hadjiiska, P. Iaydjiev, M. Rodozov, S. Stoykova, G. Sultanov, M. Vutova, A. Dimitrov, I. Glushkov, L. Litov, B. Pavlov, P. Petkov, M. Ahmad, J. G. Bian, G. M. Chen, H. S. Chen, M. Chen, T. Cheng, R. Du, C. H. Jiang, D. Leggat, R. Plestina, F. Romeo, S. M. Shaheen, A. Spiezia, J. Tao, C. Wang, Z. Wang, H. Zhang, C. Asawatangtrakuldee, Y. Ban, Q. Li, S. Liu, Y. Mao, S. J. Qian, D. Wang, Z. Xu, C. Avila, A. Cabrera, L. F. Chaparro Sierra, C. Florez, J. P. Gomez, B. Gomez Moreno, J. C. Sanabria, N. Godinovic, D. Lelas, I. Puljak, P. M. Ribeiro Cipriano, Z. Antunovic, M. Kovac, V. Brigljevic, K. Kadija, J. Luetic, S. Micanovic, L. Sudic, A. Attikis, G. Mavromanolakis, J. Mousa, C. Nicolaou, F. Ptochos, P. A. Razis, H. Rykaczewski, M. Bodlak, M. Finger, M. Finger, Y. Assran, S. Elgammal, A. Ellithi Kamel, M. A. Mahmoud, B. Calpas, M. Kadastik, M. Murumaa, M. Raidal, A. Tiko, C. Veelken, P. Eerola, J. Pekkanen, M. Voutilainen, J. Härkönen, V. Karimäki, R. Kinnunen, T. Lampén, K. Lassila-Perini, S. Lehti, T. Lindén, P. Luukka, T. Peltola, J. Tuominiemi, E. Tuovinen, L. Wendland, J. Talvitie, T. Tuuva, M. Besancon, F. Couderc, M. Dejardin, D. Denegri, B. Fabbro, J. L. Faure, C. Favaro, F. Ferri, S. Ganjour, A. Givernaud, P. Gras, G. Hamel de Monchenault, P. Jarry, E. Locci, M. Machet, J. Malcles, J. Rander, A. Rosowsky, M. Titov, A. Zghiche, A. Abdulsalam, I. Antropov, S. Baffioni, F. Beaudette, P. Busson, L. Cadamuro, E. Chapon, C. Charlot, O. Davignon, N. Filipovic, R. Granier de Cassagnac, M. Jo, S. Lisniak, L. Mastrolorenzo, P. Miné, I. N. Naranjo, M. Nguyen, C. Ochando, G. Ortona, P. Paganini, P. Pigard, S. Regnard, R. Salerno, J. B. Sauvan, Y. Sirois, T. Strebler, Y. Yilmaz, A. Zabi, J.-L. Agram, J. Andrea, A. Aubin, D. Bloch, J.-M. Brom, M. Buttignol, E. C. Chabert, N. Chanon, C. Collard, E. Conte, X. Coubez, J.-C. Fontaine, D. Gelé, U. Goerlach, C. Goetzmann, A.-C. Le Bihan, J. A. Merlin, K. Skovpen, P. Van Hove, S. Gadrat, S. Beauceron, C. Bernet, G. Boudoul, E. Bouvier, C. A. Carrillo Montoya, R. Chierici, D. Contardo, B. Courbon, P. Depasse, H. El Mamouni, J. Fan, J. Fay, S. Gascon, M. Gouzevitch, B. Ille, F. Lagarde, I. B. Laktineh, M. Lethuillier, L. Mirabito, A. L. Pequegnot, S. Perries, J. D. Ruiz Alvarez, D. Sabes, L. Sgandurra, V. Sordini, M. Vander Donckt, P. Verdier, S. Viret, T. Toriashvili, L. Rurua, C. Autermann, S. Beranek, L. Feld, A. Heister, M. K. Kiesel, K. Klein, M. Lipinski, A. Ostapchuk, M. Preuten, F. Raupach, S. Schael, J. F. Schulte, T. Verlage, H. Weber, V. Zhukov, M. Ata, M. Brodski, E. Dietz-Laursonn, D. Duchardt, M. Endres, M. Erdmann, S. Erdweg, T. Esch, R. Fischer, A. Güth, T. Hebbeker, C. Heidemann, K. Hoepfner, S. Knutzen, P. Kreuzer, M. Merschmeyer, A. Meyer, P. Millet, S. Mukherjee, M. Olschewski, K. Padeken, P. Papacz, T. Pook, M. Radziej, H. Reithler, M. Rieger, F. Scheuch, L. Sonnenschein, D. Teyssier, S. Thüer, V. Cherepanov, Y. Erdogan, G. Flügge, H. Geenen, M. Geisler, F. Hoehle, B. Kargoll, T. Kress, A. Künsken, J. Lingemann, A. Nehrkorn, A. Nowack, I. M. Nugent, C. Pistone, O. Pooth, A. Stahl, M. Aldaya Martin, I. Asin, N. Bartosik, O. Behnke, U. Behrens, K. Borras, A. Burgmeier, A. Campbell, C. Contreras-Campana, F. Costanza, C. Diez Pardos, G. Dolinska, S. Dooling, T. Dorland, G. Eckerlin, D. Eckstein, T. Eichhorn, G. Flucke, E. Gallo, J. Garay Garcia, A. Geiser, A. Gizhko, P. Gunnellini, J. Hauk, M. Hempel, H. Jung, A. Kalogeropoulos, O. Karacheban, M. Kasemann, P. Katsas, J. Kieseler, C. Kleinwort, I. Korol, W. Lange, J. Leonard, K. Lipka, A. Lobanov, W. Lohmann, R. Mankel, I.-A. Melzer-Pellmann, A. B. Meyer, G. Mittag, J. Mnich, A. Mussgiller, S. Naumann-Emme, A. Nayak, E. Ntomari, H. Perrey, D. Pitzl, R. Placakyte, A. Raspereza, B. Roland, M. Ö. Sahin, P. Saxena, T. Schoerner-Sadenius, C. Seitz, S. Spannagel, N. Stefaniuk, K. D. Trippkewitz, R. Walsh, C. Wissing, V. Blobel, M. Centis Vignali, A. R. Draeger, J. Erfle, E. Garutti, K. Goebel, D. Gonzalez, M. Görner, J. Haller, M. Hoffmann, R. S. Höing, A. Junkes, R. Klanner, R. Kogler, N. Kovalchuk, T. Lapsien, T. Lenz, I. Marchesini, D. Marconi, M. Meyer, D. Nowatschin, J. Ott, F. Pantaleo, T. Peiffer, A. Perieanu, N. Pietsch, J. Poehlsen, D. Rathjens, C. Sander, C. Scharf, P. Schleper, E. Schlieckau, A. Schmidt, S. Schumann, J. Schwandt, V. Sola, H. Stadie, G. Steinbrück, F. M. Stober, H. Tholen, D. Troendle, E. Usai, L. Vanelderen, A. Vanhoefer, B. Vormwald, C. Barth, C. Baus, J. Berger, C. Böser, E. Butz, T. Chwalek, F. Colombo, W. De Boer, A. Descroix, A. Dierlamm, S. Fink, F. Frensch, R. Friese, M. Giffels, A. Gilbert, D. Haitz, F. Hartmann, S. M. Heindl, U. Husemann, I. Katkov, A. Kornmayer, P. Lobelle Pardo, B. Maier, H. Mildner, M. U. Mozer, T. Müller, Th. Müller, M. Plagge, G. Quast, K. Rabbertz, S. Röcker, F. Roscher, M. Schröder, G. Sieber, H. J. Simonis, R. Ulrich, J. Wagner-Kuhr, S. Wayand, M. Weber, T. Weiler, S. Williamson, C. Wöhrmann, R. Wolf, G. Anagnostou, G. Daskalakis, T. Geralis, V. A. Giakoumopoulou, A. Kyriakis, D. Loukas, A. Psallidas, I. Topsis-Giotis, A. Agapitos, S. Kesisoglou, A. Panagiotou, N. Saoulidou, E. Tziaferi, I. Evangelou, G. Flouris, C. Foudas, P. Kokkas, N. Loukas, N. Manthos, I. Papadopoulos, E. Paradas, J. Strologas, G. Bencze, C. Hajdu, A. Hazi, P. Hidas, D. Horvath, F. Sikler, V. Veszpremi, G. Vesztergombi, A. J. Zsigmond, N. Beni, S. Czellar, J. Karancsi, J. Molnar, Z. Szillasi, M. Bartók, A. Makovec, P. Raics, Z. L. Trocsanyi, B. Ujvari, S. Choudhury, P. Mal, K. Mandal, D. K. Sahoo, N. Sahoo, S. K. Swain, S. Bansal, S. B. Beri, V. Bhatnagar, R. Chawla, R. Gupta, U. Bhawandeep, A. K. Kalsi, A. Kaur, M. Kaur, R. Kumar, A. Mehta, M. Mittal, J. B. Singh, G. Walia, Ashok Kumar, A. Bhardwaj, B. C. Choudhary, R. B. Garg, S. Malhotra, M. Naimuddin, N. Nishu, K. Ranjan, R. Sharma, V. Sharma, S. Bhattacharya, K. Chatterjee, S. Dey, S. Dutta, N. Majumdar, A. Modak, K. Mondal, S. Mukhopadhyay, A. Roy, D. Roy, S. Roy Chowdhury, S. Sarkar, M. Sharan, R. Chudasama, D. Dutta, V. Jha, V. Kumar, A. K. Mohanty, L. M. Pant, P. Shukla, A. Topkar, T. Aziz, S. Banerjee, S. Bhowmik, R. M. Chatterjee, R. K. Dewanjee, S. Dugad, S. Ganguly, S. Ghosh, M. Guchait, A. Gurtu, Sa. Jain, G. Kole, S. Kumar, B. Mahakud, M. Maity, G. Majumder, K. Mazumdar, S. Mitra, G. B. Mohanty, B. Parida, T. Sarkar, N. Sur, B. Sutar, N. Wickramage, S. Chauhan, S. Dube, A. Kapoor, K. Kothekar, S. Sharma, H. Bakhshiansohi, H. Behnamian, S. M. Etesami, A. Fahim, M. Khakzad, M. Mohammadi Najafabadi, M. Naseri, S. Paktinat Mehdiabadi, F. Rezaei Hosseinabadi, B. Safarzadeh, M. Zeinali, M. Felcini, M. Grunewald, M. Abbrescia, C. Calabria, C. Caputo, A. Colaleo, D. Creanza, L. Cristella, N. De Filippis, M. De Palma, L. Fiore, G. Iaselli, G. Maggi, M. Maggi, G. Miniello, S. My, S. Nuzzo, A. Pompili, G. Pugliese, R. Radogna, A. Ranieri, G. Selvaggi, L. Silvestris, R. Venditti, G. Abbiendi, C. Battilana, D. Bonacorsi, S. Braibant-Giacomelli, L. Brigliadori, R. Campanini, P. Capiluppi, A. Castro, F. R. Cavallo, S. S. Chhibra, G. Codispoti, M. Cuffiani, G. M. Dallavalle, F. Fabbri, A. Fanfani, D. Fasanella, P. Giacomelli, C. Grandi, L. Guiducci, S. Marcellini, G. Masetti, A. Montanari, F. L. Navarria, A. Perrotta, A. M. Rossi, T. Rovelli, G. P. Siroli, N. Tosi, G. Cappello, M. Chiorboli, S. Costa, A. Di Mattia, F. Giordano, R. Potenza, A. Tricomi, C. Tuve, G. Barbagli, V. Ciulli, C. Civinini, R. D’Alessandro, E. Focardi, V. Gori, P. Lenzi, M. Meschini, S. Paoletti, G. Sguazzoni, L. Viliani, L. Benussi, S. Bianco, F. Fabbri, D. Piccolo, F. Primavera, V. Calvelli, F. Ferro, M. Lo Vetere, M. R. Monge, E. Robutti, S. Tosi, L. Brianza, M. E. Dinardo, S. Fiorendi, S. Gennai, R. Gerosa, A. Ghezzi, P. Govoni, S. Malvezzi, R. A. Manzoni, B. Marzocchi, D. Menasce, L. Moroni, M. Paganoni, D. Pedrini, S. Ragazzi, N. Redaelli, T. Tabarelli de Fatis, S. Buontempo, N. Cavallo, S. Di Guida, M. Esposito, F. Fabozzi, A. O. M. Iorio, G. Lanza, L. Lista, S. Meola, M. Merola, P. Paolucci, C. Sciacca, F. Thyssen, P. Azzi, N. Bacchetta, L. Benato, D. Bisello, A. Boletti, A. Branca, R. Carlin, P. Checchia, M. Dall’Osso, T. Dorigo, U. Dosselli, F. Gasparini, U. Gasparini, A. Gozzelino, K. Kanishchev, S. Lacaprara, M. Margoni, A. T. Meneguzzo, F. Montecassiano, J. Pazzini, N. Pozzobon, P. Ronchese, F. Simonetto, E. Torassa, M. Tosi, M. Zanetti, P. Zotto, A. Zucchetta, G. Zumerle, A. Braghieri, A. Magnani, P. Montagna, S. P. Ratti, V. Re, C. Riccardi, P. Salvini, I. Vai, P. Vitulo, L. Alunni Solestizi, G. M. Bilei, D. Ciangottini, L. Fanò, P. Lariccia, G. Mantovani, M. Menichelli, A. Saha, A. Santocchia, K. Androsov, P. Azzurri, G. Bagliesi, J. Bernardini, T. Boccali, R. Castaldi, M. A. Ciocci, R. Dell’Orso, S. Donato, G. Fedi, L. Foà, A. Giassi, M. T. Grippo, F. Ligabue, T. Lomtadze, L. Martini, A. Messineo, F. Palla, A. Rizzi, A. Savoy-Navarro, A. T. Serban, P. Spagnolo, R. Tenchini, G. Tonelli, A. Venturi, P. G. Verdini, L. Barone, F. Cavallari, G. D’imperio, D. Del Re, M. Diemoz, S. Gelli, C. Jorda, E. Longo, F. Margaroli, P. Meridiani, G. Organtini, R. Paramatti, F. Preiato, S. Rahatlou, C. Rovelli, F. Santanastasio, P. Traczyk, N. Amapane, R. Arcidiacono, S. Argiro, M. Arneodo, R. Bellan, C. Biino, N. Cartiglia, M. Costa, R. Covarelli, P. De Remigis, A. Degano, N. Demaria, L. Finco, C. Mariotti, S. Maselli, E. Migliore, V. Monaco, E. Monteil, M. M. Obertino, L. Pacher, N. Pastrone, M. Pelliccioni, G. L. Pinna Angioni, F. Ravera, A. Romero, M. Ruspa, R. Sacchi, A. Solano, A. Staiano, S. Belforte, V. Candelise, M. Casarsa, F. Cossutti, G. Della Ricca, B. Gobbo, C. La Licata, M. Marone, A. Schizzi, A. Zanetti, A. Kropivnitskaya, S. K. Nam, D. H. Kim, G. N. Kim, M. S. Kim, D. J. Kong, S. Lee, Y. D. Oh, A. Sakharov, D. C. Son, J. A. Brochero Cifuentes, H. Kim, T. J. Kim, S. Song, S. Cho, S. Choi, Y. Go, D. Gyun, B. Hong, H. Kim, Y. Kim, B. Lee, K. Lee, K. S. Lee, S. Lee, J. Lim, S. K. Park, Y. Roh, H. D. Yoo, M. Choi, H. Kim, J. H. Kim, J. S. H. Lee, I. C. Park, G. Ryu, M. S. Ryu, Y. Choi, J. Goh, D. Kim, E. Kwon, J. Lee, I. Yu, V. Dudenas, A. Juodagalvis, J. Vaitkus, I. Ahmed, Z. A. Ibrahim, J. R. Komaragiri, M. A. B. Md Ali, F. Mohamad Idris, W. A. T. Wan Abdullah, M. N. Yusli, Z. Zolkapli, E. Casimiro Linares, H. Castilla-Valdez, E. De La Cruz-Burelo, I. Heredia-De La Cruz, A. Hernandez-Almada, R. Lopez-Fernandez, J. Mejia Guisao, A. Sanchez-Hernandez, S. Carrillo Moreno, F. Vazquez Valencia, I. Pedraza, H. A. Salazar Ibarguen, C. Uribe Estrada, A. Morelos Pineda, D. Krofcheck, P. H. Butler, A. Ahmad, M. Ahmad, Q. Hassan, H. R. Hoorani, W. A. Khan, S. Qazi, M. Shoaib, M. Waqas, H. Bialkowska, M. Bluj, B. Boimska, T. Frueboes, M. Górski, M. Kazana, K. Nawrocki, K. Romanowska-Rybinska, M. Szleper, P. Zalewski, G. Brona, K. Bunkowski, A. Byszuk, K. Doroba, A. Kalinowski, M. Konecki, J. Krolikowski, M. Misiura, M. Olszewski, M. Walczak, P. Bargassa, C. Beirão Da Cruz E Silva, A. Di Francesco, P. Faccioli, P. G. Ferreira Parracho, M. Gallinaro, J. Hollar, N. Leonardo, L. Lloret Iglesias, F. Nguyen, J. Rodrigues Antunes, J. Seixas, O. Toldaiev, D. Vadruccio, J. Varela, P. Vischia, I. Golutvin, I. Gorbunov, V. Karjavin, V. Korenkov, A. Lanev, A. Malakhov, V. Matveev, V. V. Mitsyn, P. Moisenz, V. Palichik, V. Perelygin, M. Savina, S. Shmatov, S. Shulha, N. Skatchkov, V. Smirnov, B. S. Yuldashev, A. Zarubin, V. Golovtsov, Y. Ivanov, V. Kim, E. Kuznetsova, P. Levchenko, V. Murzin, V. Oreshkin, I. Smirnov, V. Sulimov, L. Uvarov, S. Vavilov, A. Vorobyev, Yu. Andreev, A. Dermenev, S. Gninenko, N. Golubev, A. Karneyeu, M. Kirsanov, N. Krasnikov, A. Pashenkov, D. Tlisov, A. Toropin, V. Epshteyn, V. Gavrilov, N. Lychkovskaya, V. Popov, l. Pozdnyakov, G. Safronov, A. Spiridonov, E. Vlasov, A. Zhokin, M. Chadeeva, R. Chistov, M. Danilov, V. Rusinov, E. Tarkovskii, V. Andreev, M. Azarkin, I. Dremin, M. Kirakosyan, A. Leonidov, G. Mesyats, S. V. Rusakov, A. Baskakov, A. Belyaev, E. Boos, M. Dubinin, L. Dudko, A. Ershov, A. Gribushin, V. Klyukhin, O. Kodolova, I. Lokhtin, I. Miagkov, S. Obraztsov, S. Petrushanko, V. Savrin, A Snigirev, I. Azhgirey, I. Bayshev, S. Bitioukov, V. Kachanov, A. Kalinin, D. Konstantinov, V. Krychkine, V. Petrov, R. Ryutin, A. Sobol, L. Tourtchanovitch, S. Troshin, N. Tyurin, A. Uzunian, A. Volkov, P. Adzic, P. Cirkovic, D. Devetak, J. Milosevic, V. Rekovic, J. Alcaraz Maestre, E. Calvo, M. Cerrada, M. Chamizo Llatas, N. Colino, B. De La Cruz, A. Delgado Peris, A. Escalante Del Valle, C. Fernandez Bedoya, J. P. Fernández Ramos, J. Flix, M. C. Fouz, P. Garcia-Abia, O. Gonzalez Lopez, S. Goy Lopez, J. M. Hernandez, M. I. Josa, E. Navarro De Martino, A. Pérez-Calero Yzquierdo, J. Puerta Pelayo, A. Quintario Olmeda, I. Redondo, L. Romero, J. Santaolalla, M. S. Soares, C. Albajar, J. F. de Trocóniz, M. Missiroli, D. Moran, J. Cuevas, J. Fernandez Menendez, S. Folgueras, I. Gonzalez Caballero, E. Palencia Cortezon, J. M. Vizan Garcia, I. J. Cabrillo, A. Calderon, J. R. Castiñeiras De Saa, E. Curras, P. De Castro Manzano, M. Fernandez, J. Garcia-Ferrero, G. Gomez, A. Lopez Virto, J. Marco, R. Marco, C. Martinez Rivero, F. Matorras, J. Piedra Gomez, T. Rodrigo, A. Y. Rodríguez-Marrero, A. Ruiz-Jimeno, L. Scodellaro, N. Trevisani, I. Vila, R. Vilar Cortabitarte, D. Abbaneo, E. Auffray, G. Auzinger, M. Bachtis, P. Baillon, A. H. Ball, D. Barney, A. Benaglia, J. Bendavid, L. Benhabib, G. M. Berruti, P. Bloch, A. Bocci, A. Bonato, C. Botta, H. Breuker, T. Camporesi, R. Castello, G. Cerminara, M. D’Alfonso, D. d’Enterria, A. Dabrowski, V. Daponte, A. David, M. De Gruttola, F. De Guio, A. De Roeck, S. De Visscher, E. Di Marco, M. Dobson, M. Dordevic, B. Dorney, T. du Pree, D. Duggan, M. Dünser, N. Dupont, A. Elliott-Peisert, G. Franzoni, J. Fulcher, W. Funk, D. Gigi, K. Gill, D. Giordano, M. Girone, F. Glege, R. Guida, S. Gundacker, M. Guthoff, J. Hammer, P. Harris, J. Hegeman, V. Innocente, P. Janot, H. Kirschenmann, M. J. Kortelainen, K. Kousouris, K. Krajczar, P. Lecoq, C. Lourenço, M. T. Lucchini, N. Magini, L. Malgeri, M. Mannelli, A. Martelli, L. Masetti, F. Meijers, S. Mersi, E. Meschi, F. Moortgat, S. Morovic, M. Mulders, M. V. Nemallapudi, H. Neugebauer, S. Orfanelli, L. Orsini, L. Pape, E. Perez, M. Peruzzi, A. Petrilli, G. Petrucciani, A. Pfeiffer, M. Pierini, D. Piparo, A. Racz, T. Reis, G. Rolandi, M. Rovere, M. Ruan, H. Sakulin, C. Schäfer, C. Schwick, M. Seidel, A. Sharma, P. Silva, M. Simon, P. Sphicas, J. Steggemann, B. Stieger, M. Stoye, Y. Takahashi, D. Treille, A. Triossi, A. Tsirou, G. I. Veres, N. Wardle, H. K. Wöhri, A. Zagozdzinska, W. D. Zeuner, W. Bertl, K. Deiters, W. Erdmann, R. Horisberger, Q. Ingram, H. C. Kaestli, D. Kotlinski, U. Langenegger, T. Rohe, F. Bachmair, L. Bäni, L. Bianchini, B. Casal, G. Dissertori, M. Dittmar, M. Donegà, P. Eller, C. Grab, C. Heidegger, D. Hits, J. Hoss, G. Kasieczka, P. Lecomte, W. Lustermann, B. Mangano, M. Marionneau, P. Martinez Ruiz del Arbol, M. Masciovecchio, M. T. Meinhard, D. Meister, F. Micheli, P. Musella, F. Nessi-Tedaldi, F. Pandolfi, J. Pata, F. Pauss, L. Perrozzi, M. Quittnat, M. Rossini, M. Schönenberger, A. Starodumov, M. Takahashi, V. R. Tavolaro, K. Theofilatos, R. Wallny, T. K. Aarrestad, C. Amsler, L. Caminada, M. F. Canelli, V. Chiochia, A. De Cosa, C. Galloni, A. Hinzmann, T. Hreus, B. Kilminster, C. Lange, J. Ngadiuba, D. Pinna, G. Rauco, P. Robmann, D. Salerno, Y. Yang, M. Cardaci, K. H. Chen, T. H. Doan, Sh. Jain, R. Khurana, M. Konyushikhin, C. M. Kuo, W. Lin, Y. J. Lu, A. Pozdnyakov, S. S. Yu, Arun Kumar, P. Chang, Y. H. Chang, Y. Chao, K. F. Chen, P. H. Chen, C. Dietz, F. Fiori, U. Grundler, W.-S. Hou, Y. Hsiung, Y. F. Liu, R.-S. Lu, M. Miñano Moya, E. Petrakou, J. F. Tsai, Y. M. Tzeng, B. Asavapibhop, K. Kovitanggoon, G. Singh, N. Srimanobhas, N. Suwonjandee, A. Adiguzel, S. Cerci, S. Damarseckin, Z. S. Demiroglu, C. Dozen, I. Dumanoglu, S. Girgis, G. Gokbulut, Y. Guler, E. Gurpinar, I. Hos, E. E. Kangal, A. Kayis Topaksu, G. Onengut, K. Ozdemir, S. Ozturk, B. Tali, H. Topakli, C. Zorbilmez, B. Bilin, S. Bilmis, B. Isildak, G. Karapinar, M. Yalvac, M. Zeyrek, E. Gülmez, M. Kaya, O. Kaya, E. A. Yetkin, T. Yetkin, A. Cakir, K. Cankocak, S. Sen, F. I. Vardarlı, B. Grynyov, L. Levchuk, P. Sorokin, R. Aggleton, F. Ball, L. Beck, J. J. Brooke, E. Clement, D. Cussans, H. Flacher, J. Goldstein, M. Grimes, G. P. Heath, H. F. Heath, J. Jacob, L. Kreczko, C. Lucas, Z. Meng, D. M. Newbold, S. Paramesvaran, A. Poll, T. Sakuma, S. Seif El Nasr-storey, S. Senkin, D. Smith, V. J. Smith, K. W. Bell, A. Belyaev, C. Brew, R. M. Brown, L. Calligaris, D. Cieri, D. J. A. Cockerill, J. A. Coughlan, K. Harder, S. Harper, E. Olaiya, D. Petyt, C. H. Shepherd-Themistocleous, A. Thea, I. R. Tomalin, T. Williams, S. D. Worm, M. Baber, R. Bainbridge, O. Buchmuller, A. Bundock, D. Burton, S. Casasso, M. Citron, D. Colling, L. Corpe, P. Dauncey, G. Davies, A. De Wit, M. Della Negra, P. Dunne, A. Elwood, D. Futyan, G. Hall, G. Iles, R. Lane, R. Lucas, L. Lyons, A.-M. Magnan, S. Malik, J. Nash, A. Nikitenko, J. Pela, M. Pesaresi, D. M. Raymond, A. Richards, A. Rose, C. Seez, A. Tapper, K. Uchida, M. Vazquez Acosta, T. Virdee, S. C. Zenz, J. E. Cole, P. R. Hobson, A. Khan, P. Kyberd, D. Leslie, I. D. Reid, P. Symonds, L. Teodorescu, M. Turner, A. Borzou, K. Call, J. Dittmann, K. Hatakeyama, H. Liu, N. Pastika, O. Charaf, S. I. Cooper, C. Henderson, P. Rumerio, D. Arcaro, A. Avetisyan, T. Bose, D. Gastler, D. Rankin, C. Richardson, J. Rohlf, L. Sulak, D. Zou, J. Alimena, G. Benelli, E. Berry, D. Cutts, A. Ferapontov, A. Garabedian, J. Hakala, U. Heintz, O. Jesus, E. Laird, G. Landsberg, Z. Mao, M. Narain, S. Piperov, S. Sagir, R. Syarif, R. Breedon, G. Breto, M. Calderon De La Barca Sanchez, S. Chauhan, M. Chertok, J. Conway, R. Conway, P. T. Cox, R. Erbacher, G. Funk, M. Gardner, W. Ko, R. Lander, C. Mclean, M. Mulhearn, D. Pellett, J. Pilot, F. Ricci-Tam, S. Shalhout, J. Smith, M. Squires, D. Stolp, M. Tripathi, S. Wilbur, R. Yohay, R. Cousins, P. Everaerts, A. Florent, J. Hauser, M. Ignatenko, D. Saltzberg, E. Takasugi, V. Valuev, M. Weber, K. Burt, R. Clare, J. Ellison, J. W. Gary, G. Hanson, J. Heilman, M. Ivova PANEVA, P. Jandir, E. Kennedy, F. Lacroix, O. R. Long, M. Malberti, M. Olmedo Negrete, A. Shrinivas, H. Wei, S. Wimpenny, B. R. Yates, J. G. Branson, G. B. Cerati, S. Cittolin, R. T. D’Agnolo, M. Derdzinski, A. Holzner, R. Kelley, D. Klein, J. Letts, I. Macneill, D. Olivito, S. Padhi, M. Pieri, M. Sani, V. Sharma, S. Simon, M. Tadel, A. Vartak, S. Wasserbaech, C. Welke, F. Würthwein, A. Yagil, G. Zevi Della Porta, J. Bradmiller-Feld, C. Campagnari, A. Dishaw, V. Dutta, K. Flowers, M. Franco Sevilla, P. Geffert, C. George, F. Golf, L. Gouskos, J. Gran, J. Incandela, N. Mccoll, S. D. Mullin, J. Richman, D. Stuart, I. Suarez, C. West, J. Yoo, D. Anderson, A. Apresyan, A. Bornheim, J. Bunn, Y. Chen, J. Duarte, A. Mott, H. B. Newman, C. Pena, M. Spiropulu, J. R. Vlimant, S. Xie, R. Y. Zhu, M. B. Andrews, V. Azzolini, A. Calamba, B. Carlson, T. Ferguson, M. Paulini, J. Russ, M. Sun, H. Vogel, I. Vorobiev, J. P. Cumalat, W. T. Ford, A. Gaz, F. Jensen, A. Johnson, M. Krohn, T. Mulholland, U. Nauenberg, K. Stenson, S. R. Wagner, J. Alexander, A. Chatterjee, J. Chaves, J. Chu, S. Dittmer, N. Eggert, N. Mirman, G. Nicolas Kaufman, J. R. Patterson, A. Rinkevicius, A. Ryd, L. Skinnari, L. Soffi, W. Sun, S. M. Tan, W. D. Teo, J. Thom, J. Thompson, J. Tucker, Y. Weng, P. Wittich, S. Abdullin, M. Albrow, G. Apollinari, S. Banerjee, L. A. T. Bauerdick, A. Beretvas, J. Berryhill, P. C. Bhat, G. Bolla, K. Burkett, J. N. Butler, H. W. K. Cheung, F. Chlebana, S. Cihangir, V. D. Elvira, I. Fisk, J. Freeman, E. Gottschalk, L. Gray, D. Green, S. Grünendahl, O. Gutsche, J. Hanlon, D. Hare, R. M. Harris, S. Hasegawa, J. Hirschauer, Z. Hu, B. Jayatilaka, S. Jindariani, M. Johnson, U. Joshi, B. Klima, B. Kreis, S. Lammel, J. Lewis, J. Linacre, D. Lincoln, R. Lipton, T. Liu, R. Lopes De Sá, J. Lykken, K. Maeshima, J. M. Marraffino, S. Maruyama, D. Mason, P. McBride, P. Merkel, S. Mrenna, S. Nahn, C. Newman-Holmes, V. O’Dell, K. Pedro, O. Prokofyev, G. Rakness, E. Sexton-Kennedy, A. Soha, W. J. Spalding, L. Spiegel, S. Stoynev, N. Strobbe, L. Taylor, S. Tkaczyk, N. V. Tran, L. Uplegger, E. W. Vaandering, C. Vernieri, M. Verzocchi, R. Vidal, M. Wang, H. A. Weber, A. Whitbeck, D. Acosta, P. Avery, P. Bortignon, D. Bourilkov, A. Brinkerhoff, A. Carnes, M. Carver, D. Curry, S. Das, R. D. Field, I. K. Furic, J. Konigsberg, A. Korytov, K. Kotov, P. Ma, K. Matchev, H. Mei, P. Milenovic, G. Mitselmakher, D. Rank, R. Rossin, L. Shchutska, M. Snowball, D. Sperka, N. Terentyev, L. Thomas, J. Wang, S. Wang, J. Yelton, S. Hewamanage, S. Linn, P. Markowitz, G. Martinez, J. L. Rodriguez, A. Ackert, J. R. Adams, T. Adams, A. Askew, S. Bein, J. Bochenek, B. Diamond, J. Haas, S. Hagopian, V. Hagopian, K. F. Johnson, A. Khatiwada, H. Prosper, M. Weinberg, M. M. Baarmand, V. Bhopatkar, S. Colafranceschi, M. Hohlmann, H. Kalakhety, D. Noonan, T. Roy, F. Yumiceva, M. R. Adams, L. Apanasevich, D. Berry, R. R. Betts, I. Bucinskaite, R. Cavanaugh, O. Evdokimov, L. Gauthier, C. E. Gerber, D. J. Hofman, P. Kurt, C. O’Brien, l. D. Sandoval Gonzalez, P. Turner, N. Varelas, Z. Wu, M. Zakaria, J. Zhang, B. Bilki, W. Clarida, K. Dilsiz, S. Durgut, R. P. Gandrajula, M. Haytmyradov, V. Khristenko, J.-P. Merlo, H. Mermerkaya, A. Mestvirishvili, A. Moeller, J. Nachtman, H. Ogul, Y. Onel, F. Ozok, A. Penzo, C. Snyder, E. Tiras, J. Wetzel, K. Yi, I. Anderson, B. A. Barnett, B. Blumenfeld, A. Cocoros, N. Eminizer, D. Fehling, L. Feng, A. V. Gritsan, P. Maksimovic, M. Osherson, J. Roskes, U. Sarica, M. Swartz, M. Xiao, Y. Xin, C. You, P. Baringer, A. Bean, C. Bruner, R. P. Kenny, D. Majumder, M. Malek, W. Mcbrayer, M. Murray, S. Sanders, R. Stringer, Q. Wang, A. Ivanov, K. Kaadze, S. Khalil, M. Makouski, Y. Maravin, A. Mohammadi, L. K. Saini, N. Skhirtladze, S. Toda, D. Lange, F. Rebassoo, D. Wright, C. Anelli, A. Baden, O. Baron, A. Belloni, B. Calvert, S. C. Eno, C. Ferraioli, J. A. Gomez, N. J. Hadley, S. Jabeen, R. G. Kellogg, T. Kolberg, J. Kunkle, Y. Lu, A. C. Mignerey, Y. H. Shin, A. Skuja, M. B. Tonjes, S. C. Tonwar, A. Apyan, R. Barbieri, A. Baty, R. Bi, K. Bierwagen, S. Brandt, W. Busza, I. A. Cali, Z. Demiragli, L. Di Matteo, G. Gomez Ceballos, M. Goncharov, D. Gulhan, Y. Iiyama, G. M. Innocenti, M. Klute, D. Kovalskyi, Y. S. Lai, Y.-J. Lee, A. Levin, P. D. Luckey, A. C. Marini, C. Mcginn, C. Mironov, S. Narayanan, X. Niu, C. Paus, C. Roland, G. Roland, J. Salfeld-Nebgen, G. S. F. Stephans, K. Sumorok, K. Tatar, M. Varma, D. Velicanu, J. Veverka, J. Wang, T. W. Wang, B. Wyslouch, M. Yang, V. Zhukova, A. C. Benvenuti, B. Dahmes, A. Evans, A. Finkel, A. Gude, P. Hansen, S. Kalafut, S. C. Kao, K. Klapoetke, Y. Kubota, Z. Lesko, J. Mans, S. Nourbakhsh, N. Ruckstuhl, R. Rusack, N. Tambe, J. Turkewitz, J. G. Acosta, S. Oliveros, E. Avdeeva, R. Bartek, K. Bloom, S. Bose, D. R. Claes, A. Dominguez, C. Fangmeier, R. Gonzalez Suarez, R. Kamalieddin, D. Knowlton, I. Kravchenko, F. Meier, J. Monroy, F. Ratnikov, J. E. Siado, G. R. Snow, M. Alyari, J. Dolen, J. George, A. Godshalk, C. Harrington, I. Iashvili, J. Kaisen, A. Kharchilava, A. Kumar, S. Rappoccio, B. Roozbahani, G. Alverson, E. Barberis, D. Baumgartel, M. Chasco, A. Hortiangtham, A. Massironi, D. M. Morse, D. Nash, T. Orimoto, R. Teixeira De Lima, D. Trocino, R.-J. Wang, D. Wood, J. Zhang, S. Bhattacharya, K. A. Hahn, A. Kubik, J. F. Low, N. Mucia, N. Odell, B. Pollack, M. Schmitt, K. Sung, M. Trovato, M. Velasco, N. Dev, M. Hildreth, C. Jessop, D. J. Karmgard, N. Kellams, K. Lannon, N. Marinelli, F. Meng, C. Mueller, Y. Musienko, M. Planer, A. Reinsvold, R. Ruchti, G. Smith, S. Taroni, N. Valls, M. Wayne, M. Wolf, A. Woodard, L. Antonelli, J. Brinson, B. Bylsma, L. S. Durkin, S. Flowers, A. Hart, C. Hill, R. Hughes, W. Ji, T. Y. Ling, B. Liu, W. Luo, D. Puigh, M. Rodenburg, B. L. Winer, H. W. Wulsin, O. Driga, P. Elmer, J. Hardenbrook, P. Hebda, S. A. Koay, P. Lujan, D. Marlow, T. Medvedeva, M. Mooney, J. Olsen, C. Palmer, P. Piroué, D. Stickland, C. Tully, A. Zuranski, S. Malik, A. Barker, V. E. Barnes, D. Benedetti, D. Bortoletto, L. Gutay, M. K. Jha, M. Jones, A. W. Jung, K. Jung, A. Kumar, D. H. Miller, N. Neumeister, B. C. Radburn-Smith, X. Shi, I. Shipsey, D. Silvers, J. Sun, A. Svyatkovskiy, F. Wang, W. Xie, L. Xu, N. Parashar, J. Stupak, A. Adair, B. Akgun, Z. Chen, K. M. Ecklund, F. J. M. Geurts, M. Guilbaud, W. Li, B. Michlin, M. Northup, B. P. Padley, R. Redjimi, J. Roberts, J. Rorie, Z. Tu, J. Zabel, B. Betchart, A. Bodek, P. de Barbaro, R. Demina, Y. Eshaq, T. Ferbel, M. Galanti, A. Garcia-Bellido, J. Han, O. Hindrichs, A. Khukhunaishvili, K. H. Lo, P. Tan, M. Verzetti, J. P. Chou, E. Contreras-Campana, D. Ferencek, Y. Gershtein, E. Halkiadakis, M. Heindl, D. Hidas, E. Hughes, S. Kaplan, R. Kunnawalkam Elayavalli, A. Lath, K. Nash, H. Saka, S. Salur, S. Schnetzer, D. Sheffield, S. Somalwar, R. Stone, S. Thomas, P. Thomassen, M. Walker, M. Foerster, G. Riley, K. Rose, S. Spanier, K. Thapa, O. Bouhali, A. Castaneda Hernandez, A. Celik, M. Dalchenko, M. De Mattia, A. Delgado, S. Dildick, R. Eusebi, J. Gilmore, T. Huang, T. Kamon, V. Krutelyov, R. Mueller, I. Osipenkov, Y. Pakhotin, R. Patel, A. Perloff, A. Rose, A. Safonov, A. Tatarinov, K. A. Ulmer, N. Akchurin, C. Cowden, J. Damgov, C. Dragoiu, P. R. Dudero, J. Faulkner, S. Kunori, K. Lamichhane, S. W. Lee, T. Libeiro, S. Undleeb, I. Volobouev, E. Appelt, A. G. Delannoy, S. Greene, A. Gurrola, R. Janjam, W. Johns, C. Maguire, Y. Mao, A. Melo, H. Ni, P. Sheldon, S. Tuo, J. Velkovska, Q. Xu, M. W. Arenton, B. Cox, B. Francis, J. Goodell, R. Hirosky, A. Ledovskoy, H. Li, C. Lin, C. Neu, T. Sinthuprasith, X. Sun, Y. Wang, E. Wolfe, J. Wood, F. Xia, C. Clarke, R. Harr, P. E. Karchin, C. Kottachchi Kankanamge Don, P. Lamichhane, J. Sturdy, D. A. Belknap, D. Carlsmith, M. Cepeda, S. Dasu, L. Dodd, S. Duric, B. Gomber, M. Grothe, M. Herndon, A. Hervé, P. Klabbers, A. Lanaro, A. Levine, K. Long, R. Loveless, A. Mohapatra, I. Ojalvo, T. Perry, G. A. Pierro, G. Polese, T. Ruggles, T. Sarangi, A. Savin, A. Sharma, N. Smith, W. H. Smith, D. Taylor, P. Verwilligen, N. Woods, [Authorinst]The CMS Collaboration

**Affiliations:** 1 Yerevan Physics Institute, Yerevan, Armenia; 2 Institut für Hochenergiephysik der OeAW, Vienna, Austria; 3 National Centre for Particle and High Energy Physics, Minsk, Belarus; 4 Universiteit Antwerpen, Antwerp, Belgium; 5 Vrije Universiteit Brussel, Brussels, Belgium; 6 Université Libre de Bruxelles, Brussels, Belgium; 7 Ghent University, Ghent, Belgium; 8 Université Catholique de Louvain, Louvain-la-Neuve, Belgium; 9 Université de Mons, Mons, Belgium; 10 Centro Brasileiro de Pesquisas Fisicas, Rio de Janeiro, Brazil; 11 Universidade do Estado do Rio de Janeiro, Rio de Janeiro, Brazil; 12 Universidade Estadual Paulista, Universidade Federal do ABC, São Paulo, Brazil; 13 Institute for Nuclear Research and Nuclear Energy, Sofia, Bulgaria; 14 University of Sofia, Sofia, Bulgaria; 15 Institute of High Energy Physics, Beijing, China; 16 State Key Laboratory of Nuclear Physics and Technology, Peking University, Beijing, China; 17 Universidad de Los Andes, Bogotá, Colombia; 18 Faculty of Electrical Engineering, Mechanical Engineering and Naval Architecture, University of Split, Split, Croatia; 19 Faculty of Science, University of Split, Split, Croatia; 20 Institute Rudjer Boskovic, Zagreb, Croatia; 21 University of Cyprus, Nicosia, Cyprus; 22 Charles University, Prague, Czech Republic; 23 Academy of Scientific Research and Technology of the Arab Republic of Egypt, Egyptian Network of High Energy Physics, Cairo, Egypt; 24 National Institute of Chemical Physics and Biophysics, Tallinn, Estonia; 25 Department of Physics, University of Helsinki, Helsinki, Finland; 26 Helsinki Institute of Physics, Helsinki, Finland; 27 Lappeenranta University of Technology, Lappeenranta, Finland; 28 DSM/IRFU, CEA/Saclay, Gif-sur-Yvette, France; 29 Laboratoire Leprince-Ringuet, Ecole Polytechnique, IN2P3-CNRS, Palaiseau, France; 30 Institut Pluridisciplinaire Hubert Curien, Université de Strasbourg, Université de Haute Alsace Mulhouse, CNRS/IN2P3, Strasbourg, France; 31 Centre de Calcul de l’Institut National de Physique Nucleaire et de Physique des Particules, CNRS/IN2P3, Villeurbanne, France; 32 Institut de Physique Nucléaire de Lyon, Université de Lyon, Université Claude Bernard Lyon 1, CNRS-IN2P3, Villeurbanne, France; 33 Georgian Technical University, Tbilisi, Georgia; 34 Tbilisi State University, Tbilisi, Georgia; 35 I. Physikalisches Institut, RWTH Aachen University, Aachen, Germany; 36 III. Physikalisches Institut A, RWTH Aachen University, Aachen, Germany; 37 III. Physikalisches Institut B, RWTH Aachen University, Aachen, Germany; 38 Deutsches Elektronen-Synchrotron, Hamburg, Germany; 39 University of Hamburg, Hamburg, Germany; 40 Institut für Experimentelle Kernphysik, Karlsruhe, Germany; 41 Institute of Nuclear and Particle Physics (INPP), NCSR Demokritos, Aghia Paraskevi, Greece; 42 National and Kapodistrian University of Athens, Athens, Greece; 43 University of Ioánnina, Ioánnina, Greece; 44 Wigner Research Centre for Physics, Budapest, Hungary; 45 Institute of Nuclear Research ATOMKI, Debrecen, Hungary; 46 University of Debrecen, Debrecen, Hungary; 47 National Institute of Science Education and Research, Bhubaneswar, India; 48 Panjab University, Chandigarh, India; 49 University of Delhi, Delhi, India; 50 Saha Institute of Nuclear Physics, Kolkata, India; 51 Bhabha Atomic Research Centre, Mumbai, India; 52 Tata Institute of Fundamental Research, Mumbai, India; 53 Indian Institute of Science Education and Research (IISER), Pune, India; 54 Institute for Research in Fundamental Sciences (IPM), Tehran, Iran; 55 University College Dublin, Dublin, Ireland; 56 INFN Sezione di Bari, Università di Bari, Politecnico di Bari, Bari, Italy; 57 INFN Sezione di Bologna, Università di Bologna, Bologna, Italy; 58 INFN Sezione di Catania, Università di Catania, Catania, Italy; 59 INFN Sezione di Firenze, Università di Firenze, Florence, Italy; 60 INFN Laboratori Nazionali di Frascati, Frascati, Italy; 61 INFN Sezione di Genova, Università di Genova, Genoa, Italy; 62 INFN Sezione di Milano-Bicocca, Università di Milano-Bicocca, Milan, Italy; 63 INFN Sezione di Napoli, Università di Napoli ‘Federico II’, Naples, Italy, Università della Basilicata, Potenza, Italy, Università G. Marconi, Rome, Italy; 64 INFN Sezione di Padova, Università di Padova, Padova, Italy, Università di Trento, Trento, Italy; 65 INFN Sezione di Pavia, Università di Pavia, Pavia, Italy; 66 INFN Sezione di Perugia, Università di Perugia, Perugia, Italy; 67 INFN Sezione di Pisa, Università di Pisa, Scuola Normale Superiore di Pisa, Pisa, Italy; 68 INFN Sezione di Roma, Università di Roma, Rome, Italy; 69 INFN Sezione di Torino, Università di Torino, Turin, Italy, Università del Piemonte Orientale, Novara, Italy; 70 INFN Sezione di Trieste, Università di Trieste, Trieste, Italy; 71 Kangwon National University, Chunchon, Korea; 72 Kyungpook National University, Daegu, Korea; 73 Chonbuk National University, Jeonju, Korea; 74 Institute for Universe and Elementary Particles, Chonnam National University, Kwangju, Korea; 75 Korea University, Seoul, Korea; 76 Seoul National University, Seoul, Korea; 77 University of Seoul, Seoul, Korea; 78 Sungkyunkwan University, Suwon, Korea; 79 Vilnius University, Vilnius, Lithuania; 80 National Centre for Particle Physics, Universiti Malaya, Kuala Lumpur, Malaysia; 81 Centro de Investigacion y de Estudios Avanzados del IPN, Mexico City, Mexico; 82 Universidad Iberoamericana, Mexico City, Mexico; 83 Benemerita Universidad Autonoma de Puebla, Puebla, Mexico; 84 Universidad Autónoma de San Luis Potosí, San Luis Potosí, Mexico; 85 University of Auckland, Auckland, New Zealand; 86 University of Canterbury, Christchurch, New Zealand; 87 National Centre for Physics, Quaid-I-Azam University, Islamabad, Pakistan; 88 National Centre for Nuclear Research, Swierk, Poland; 89 Institute of Experimental Physics, Faculty of Physics, University of Warsaw, Warsaw, Poland; 90 Laboratório de Instrumentação e Física Experimental de Partículas, Lisbon, Portugal; 91 Joint Institute for Nuclear Research, Dubna, Russia; 92 Petersburg Nuclear Physics Institute, Gatchina, St. Petersburg, Russia; 93 Institute for Nuclear Research, Moscow, Russia; 94 Institute for Theoretical and Experimental Physics, Moscow, Russia; 95 National Research Nuclear University ‘Moscow Engineering Physics Institute’ (MEPhI), Moscow, Russia; 96 P. N. Lebedev Physical Institute, Moscow, Russia; 97 Skobeltsyn Institute of Nuclear Physics, Lomonosov Moscow State University, Moscow, Russia; 98 State Research Center of Russian Federation, Institute for High Energy Physics, Protvino, Russia; 99 Faculty of Physics and Vinca Institute of Nuclear Sciences, University of Belgrade, Belgrade, Serbia; 100 Centro de Investigaciones Energéticas Medioambientales y Tecnológicas (CIEMAT), Madrid, Spain; 101 Universidad Autónoma de Madrid, Madrid, Spain; 102 Universidad de Oviedo, Oviedo, Spain; 103 Instituto de Física de Cantabria (IFCA), CSIC-Universidad de Cantabria, Santander, Spain; 104 CERN, European Organization for Nuclear Research, Geneva, Switzerland; 105 Paul Scherrer Institut, Villigen, Switzerland; 106 Institute for Particle Physics, ETH Zurich, Zurich, Switzerland; 107 Universität Zürich, Zurich, Switzerland; 108 National Central University, Chung-Li, Taiwan; 109 National Taiwan University (NTU), Taipei, Taiwan; 110 Department of Physics, Faculty of Science, Chulalongkorn University, Bangkok, Thailand; 111 Cukurova University, Adana, Turkey; 112 Physics Department, Middle East Technical University, Ankara, Turkey; 113 Bogazici University, Istanbul, Turkey; 114 Istanbul Technical University, Istanbul, Turkey; 115 Institute for Scintillation Materials of National Academy of Science of Ukraine, Kharkov, Ukraine; 116 National Scientific Center, Kharkov Institute of Physics and Technology, Kharkov, Ukraine; 117 University of Bristol, Bristol, UK; 118 Rutherford Appleton Laboratory, Didcot, UK; 119 Imperial College, London, UK; 120 Brunel University, Uxbridge, UK; 121 Baylor University, Waco, USA; 122 The University of Alabama, Tuscaloosa, USA; 123 Boston University, Boston, USA; 124 Brown University, Providence, USA; 125 University of California, Davis, Davis, USA; 126 University of California, Los Angeles, USA; 127 University of California, Riverside, Riverside, USA; 128 University of California, San Diego, La Jolla, USA; 129 University of California, Santa Barbara, Santa Barbara, USA; 130 California Institute of Technology, Pasadena, USA; 131 Carnegie Mellon University, Pittsburgh, USA; 132 University of Colorado Boulder, Boulder, USA; 133 Cornell University, Ithaca, USA; 134 Fermi National Accelerator Laboratory, Batavia, USA; 135 University of Florida, Gainesville, USA; 136 Florida International University, Miami, USA; 137 Florida State University, Tallahassee, USA; 138 Florida Institute of Technology, Melbourne, USA; 139 University of Illinois at Chicago (UIC), Chicago, USA; 140 The University of Iowa, Iowa City, USA; 141 Johns Hopkins University, Baltimore, USA; 142 The University of Kansas, Lawrence, USA; 143 Kansas State University, Manhattan, USA; 144 Lawrence Livermore National Laboratory, Livermore, USA; 145 University of Maryland, College Park, USA; 146 Massachusetts Institute of Technology, Cambridge, USA; 147 University of Minnesota, Minneapolis, USA; 148 University of Mississippi, Oxford, USA; 149 University of Nebraska-Lincoln, Lincoln, USA; 150 State University of New York at Buffalo, Buffalo, USA; 151 Northeastern University, Boston, USA; 152 Northwestern University, Evanston, USA; 153 University of Notre Dame, Notre Dame, USA; 154 The Ohio State University, Columbus, USA; 155 Princeton University, Princeton, USA; 156 University of Puerto Rico, Mayaguez, USA; 157 Purdue University, West Lafayette, USA; 158 Purdue University Calumet, Hammond, USA; 159 Rice University, Houston, USA; 160 University of Rochester, Rochester, USA; 161 Rutgers, The State University of New Jersey, Piscataway, USA; 162 University of Tennessee, Knoxville, USA; 163 Texas A&M University, College Station, USA; 164 Texas Tech University, Lubbock, USA; 165 Vanderbilt University, Nashville, USA; 166 University of Virginia, Charlottesville, USA; 167 Wayne State University, Detroit, USA; 168 University of Wisconsin-Madison, Madison, WI USA; 169 CERN, Geneva, Switzerland

## Abstract

A search for narrow resonances decaying to an electron and a muon is presented. The $\mathrm {e}$ ${\mu }$ mass spectrum is also investigated for non-resonant contributions from the production of quantum black holes (QBHs). The analysis is performed using data corresponding to an integrated luminosity of 19.7$~\text {fb}^\text {-1}$ collected in proton-proton collisions at a centre-of-mass energy of 8$~\text {TeV}$ with the CMS detector at the LHC. With no evidence for physics beyond the standard model in the invariant mass spectrum of selected $\mathrm {e}\mu$ pairs, upper limits are set at 95 $\%$ confidence level on the product of cross section and branching fraction for signals arising in theories with charged lepton flavour violation. In the search for narrow resonances, the resonant production of a $\mathrm {\tau }$ sneutrino in R-parity violating supersymmetry is considered. The $\mathrm {\tau }$ sneutrino is excluded for masses below 1.28$~\text {TeV}$ for couplings $\lambda _{132}=\lambda _{231}=\lambda '_{311}=0.01$, and below 2.30$~\text {TeV}$ for $\lambda _{132}=\lambda _{231}=0.07$ and $\lambda '_{311}=0.11$. These are the most stringent limits to date from direct searches at high-energy colliders. In addition, the resonance searches are interpreted in terms of a model with heavy partners of the ${\mathrm {Z}}$ boson and the photon. In a framework of TeV-scale quantum gravity based on a renormalization of Newton’s constant, the search for non-resonant contributions to the $\mathrm {e}$ ${\mu }$ mass spectrum excludes QBH production below a threshold mass $M_{\mathrm {th}}$ of 1.99$~\text {TeV}$. In models that invoke extra dimensions, the bounds range from 2.36$~\text {TeV}$ for one extra dimension to 3.63$~\text {TeV}$ for six extra dimensions. This is the first search for QBHs decaying into the $\mathrm {e}$ ${\mu }$ final state.

## Introduction

Several extensions of the standard model (SM) predict the existence of heavy, short-lived states that decay to the $\mathrm {e}$ ${\mu }$ final state, and motivate the search for lepton flavour violating (LFV) signatures in interactions involving charged leptons. This paper reports a search for phenomena beyond the SM in the invariant mass spectrum of $\mathrm {e}$ ${\mu }$ pairs. The analysis is based on data with an integrated luminosity of 19.7$~\text {fb}^\text {-1}$ collected in proton-proton ($\mathrm {p}\mathrm {p}$) collisions at $\sqrt{s} = 8~\text {TeV}$ with the CMS detector at the CERN LHC [1]. The results are interpreted in terms of three theoretically predicted objects: a $\mathrm {\tau }$ sneutrino ($\tilde{\nu }_{\mathrm {\tau }}$) lightest supersymmetric particle (LSP) in R-parity violating (RPV) supersymmetry (SUSY) [2], interfering LFV ${\mathrm {Z}}^\prime$ and $\gamma {}^\prime$ bosons [3], and quantum black holes (QBHs) [4–6].

In RPV SUSY, lepton number can be violated at tree level in interactions between fermions and sfermions, and the $\tilde{\nu }_{\mathrm {\tau }}$ may be the LSP [7]. For the resonant $\tilde{\nu }_{\mathrm {\tau }}$ signal, the following trilinear RPV part of the superpotential is considered: ${W_{\mathrm {RPV}}= \frac{1}{2} \lambda _{ijk}L_{i} L_{j} \bar{E}_{k} + \lambda '_{ijk} L_{i} Q_{j} \bar{D}_{k}}$ , where *i*, *j*, and $k \in \{1,2,3\}$ are generation indices, *L* and *Q* are the $SU(2)_{L}$ doublet superfields of the leptons and quarks, and $\bar{E}$ and $\bar{D}$ are the $SU(2)_{L}$ singlet superfields of the charged leptons and down-like quarks. We assume that all RPV couplings vanish, except for $\lambda _{132}$, $\lambda _{231}$, and $\lambda '_{311}$, and consider a SUSY mass hierarchy with a $\tilde{\nu }_{\mathrm {\tau }}$ LSP. In this model, the $\tilde{\nu }_{\mathrm {\tau }}$ can be produced resonantly in $\mathrm {p}\mathrm {p}$ collisions via the $\lambda '_{311}$ coupling and it can decay either into an $\mathrm {e}$ ${\mu }$ pair via the $\lambda _{132}$ and $\lambda _{231}$ couplings, or into a $\mathrm{d}$ $\overline{\mathrm{d}}$ pair via the $\lambda '_{311}$ coupling. In this analysis we consider only the $\mathrm {e}$ ${\mu }$ final state and, for simplicity, we assume ${\lambda _{132} =\lambda _{231}}$.

The LFV ${\mathrm {Z}}^\prime$ signal is based on a model with two extra dimensions [3, 8], where the three generations of the SM arise from a single generation in higher-dimensional space-time. Flavour changing processes are introduced through the Kaluza–Klein modes of gauge fields that are not localised on a brane. In four-dimensional space-time, an effective Lagrangian can be obtained that contains two complex vector fields ${\mathrm {Z}}^\prime$ and $\gamma {}^\prime$. These vector fields generate transitions between the families in which the generation number changes by unity, such as the process

$$\begin{aligned} {\mathrm{d}+ \overline{\mathrm{s}}\rightarrow {\mathrm {Z}}^\prime {}/\gamma {}^\prime \rightarrow \mathrm {e}^{-} + {\mu }^{+}} \end{aligned}$$

and its charge conjugate. The structure of the terms in the Lagrangian for the production and decay of the ${\mathrm {Z}}^\prime$ and $\gamma {}^\prime$ bosons is analogous to that describing the interactions of the ${\mathrm {Z}}$ boson and the photon with quarks and charged leptons, respectively. The coupling strengths $\text{ g }_{12}$ and $\text{ e }_{12}$ are related to their SM counterparts through a multiplicative coupling modifier $\kappa$. For simplicity, the masses $M_{{\mathrm {Z}}^\prime {}}$ and $M_{\gamma {}^\prime {}}$ are assumed to be equal, and the model is referred to as the LFV ${\mathrm {Z}}^\prime {}$ model. It is characterized by the two independent parameters $M_{{\mathrm {Z}}^\prime {}}$ and $\kappa$.

Theories that have a fundamental Planck scale of the order of a TeV [9–13] offer the possibility of producing microscopic black holes [14–16] at the LHC. In contrast to semiclassical, thermal black holes, which would decay to high-multiplicity final states, QBHs are non-thermal objects expected to decay predominantly to pairs of particles. We consider the production of a ${\text{ spin- }0}$, colourless, neutral QBH in a model with lepton flavour violation, in which the cross section for QBH production is extrapolated from semiclassical black holes and depends on the threshold mass $M_{\mathrm {th}}$ for QBH production and the number of extra dimensions *n*. For ${n=0}$, it corresponds to a 3+1-dimensional model with low-scale quantum gravity, where a renormalization of Newton’s constant leads to a Planck scale at the TeV scale [13, 17, 18]; ${n=1}$ corresponds to the Randall–Sundrum (RS) brane world model [9, 10]; and ${n>1}$ to the Arkani-Hamed–Dimopoulos–Dvali (ADD) model [11, 12]. We consider flat-space black holes (black holes that are spherical both in the brane and in the bulk dimensions) and, in the case of RS-type black holes ($n=1$), consider only the regime in which almost flat five-dimensional space is an applicable metric. This is the case for $r_S \ll 1/(ke^{-kr_c})$, where $r_S$ is the Schwarzschild radius, *k* denotes the Anti-de Sitter curvature, and $r_c$ is the size of the extra dimension. The threshold $M_{\mathrm {th}}$ is assumed to be at the Planck scale in the definition of the Particle Data Group [19] for ${n=0}$ and ${n>1}$, whereas for ${n=1}$ both the PDG and RS definitions [4] are adopted. In this model, the branching fraction of QBH decays to the $\mathrm {e}^{\pm }{\mu }^{\mp }$ final state is 1.1 %, which is twice that of the dimuon or dielectron decay modes, making the $\mathrm {e}^{\pm }{\mu }^{\mp }$ signature the most promising leptonic decay channel. While the resonant $\tilde{\nu }_{\mathrm {\tau }}$ and LFV ${\mathrm {Z}}^\prime$ signals result in a narrow peak in the invariant mass spectrum of the $\mathrm {e}$ ${\mu }$ pair, the mass distribution of the QBH signal is characterized by an edge at the threshold for QBH production, and a monotonically decreasing tail.

Direct searches for resonances in the $\mathrm {e}$ ${\mu }$ invariant mass spectrum with interpretations in terms of $\tilde{\nu }_{\mathrm {\tau }}$ production have been carried out by the CDF [20] and D0 [21] collaborations at the Fermilab Tevatron and most recently by the ATLAS collaboration [22] using $\mathrm {p}\mathrm {p}$ collision data at a centre-of-mass energy of $8~\text {TeV}$ at the LHC. For couplings $\lambda _{132} =0.07$ and $\lambda '_{311} =0.11$, the most stringent of these limits stems from the search performed by the ATLAS collaboration, excluding at 95 % confidence level (CL) a $\tilde{\nu }_{\mathrm {\tau }}$ below a mass of $2.0~\text {TeV}$. Low-energy muon conversion experiments [23] yield strong limits as a function of the $\mathrm {\tau }$ sneutrino mass on the product of the two RPV couplings of ${\lambda _{132} \lambda '_{311} <3.3 \times 10^{-7} \, \left( M_{\tilde{\nu }_{\mathrm {\tau }}}/1~\text {TeV} \right) ^{2}}$ at 90 % CL [24]. In the case of the ${\mathrm {Z}}^\prime$ signal, searches for ${\text{ K }^{0}_{\mathrm {L}} \rightarrow \mathrm {e}{}{\mu }{}}$ decays constrain the coupling modifier $\kappa$. For the choice $M_{{\mathrm {Z}}^\prime {}}=M_{\gamma {}^\prime {}}$, a bound of $\kappa \lesssim M_{{\mathrm {Z}}^\prime {}}/100~\text {TeV}$ is obtained at 90 % CL [3, 25]. There have been searches for QBHs decaying hadronically, by the CMS [26–28] and ATLAS [29, 30] collaborations, and in the photon plus jet, lepton plus jet, dimuon, and dielectron final states, by the ATLAS collaboration [31–34]. This is the first search for QBH decays into the $\mathrm {e}$ ${\mu }$ final state.

The search for the phenomena beyond the SM described above is carried out for invariant masses of the $\mathrm {e}$ ${\mu }$ pair of ${M_{\mathrm {e}{}{\mu }{}} \ge 200~\text {GeV}}$, which is the relevant region in light of existing constraints from other direct searches. Using the same event selection, the $\mathrm {e}$ ${\mu }$ invariant mass spectrum is searched for two different signal shapes: the shape associated with a narrow resonance that may be interpreted in terms of any model involving a resonance decaying promptly into an electron and a muon, and the more model-specific QBH signal shape. With a relative $\mathrm {e}$ ${\mu }$ invariant mass resolution ranging from 1.6 % at ${M_{\mathrm {e}{}{\mu }{}}=200~\text {GeV}}$ to 6 % at ${M_{\mathrm {e}{}{\mu }{}}=3~\text {TeV}}$, the CMS detector is a powerful tool for searches for new physics in the $\mathrm {e}$ ${\mu }$ invariant mass spectrum.

## The CMS detector

The central feature of the CMS apparatus is a superconducting solenoid of 6$\text {\,m}$ internal diameter, providing a magnetic field of 3.8$\text {\,T}$. Within the solenoid volume are a silicon pixel and strip tracker, a lead tungstate crystal electromagnetic calorimeter (ECAL), and a brass and scintillator hadron calorimeter (HCAL), each composed of a barrel and two endcap sections. Extensive forward calorimetry complements the coverage provided by the barrel and endcap detectors. Muons are measured in gas-ionization detectors embedded in the steel flux-return yoke outside the solenoid. The silicon tracker consists of 1440 silicon pixel and 15 148 silicon strip detector modules and measures charged particles within the pseudorapidity range ${|\eta |< 2.5}$. The ECAL consists of 75 848 lead tungstate crystals and provides coverage for ${| \eta |< 1.479}$ in a barrel region and ${1.479<| \eta | < 3.0}$ in two endcap regions. Muons are measured in the range ${|\eta |< 2.4}$, with detection planes using three technologies: drift tubes, cathode strip chambers, and resistive plate chambers. A two-level trigger system is used by the CMS experiment. The first level is composed of custom hardware processors and uses information from the calorimeters and muon detectors to select interesting events and to reduce the event rate from the initial bunch crossing frequency of 20$\text {\,MHz}$ to a maximum of 100$\text {\,kHz}$. The high-level trigger processor farm further decreases the event rate to 400$\text {\,Hz}$ before data storage. A detailed description of the CMS detector, together with a definition of the coordinate system used and the relevant kinematic variables, can be found in Ref. [35].

## Event selection

The search is designed in a model-independent way by requiring only one prompt, isolated muon and one prompt, isolated electron in the event selection. This minimal selection allows for a reinterpretation of the results in terms of models with more complex event topologies than the single $\mathrm {e}$ ${\mu }$ pair present in the signals considered in this paper.

The data sample is selected using a single-muon trigger with a minimum transverse momentum ($p_{\mathrm {T}}$) requirement of ${p_{\mathrm {T}} > 40~\text {GeV} {}}$. In order to allow the trigger to remain unprescaled, the pseudorapidity of the muons is constrained to values ${|\eta |<2.1}$. Offline, each event is required to have a reconstructed $\mathrm {p}\mathrm {p}$ collision vertex with at least four associated tracks, located less than 2$~\text {cm}$ from the centre of the detector in the plane transverse to the beam and less than 24$~\text {cm}$ from it in the direction along the beam. The primary vertex is defined as the vertex with the largest sum of squared transverse momenta of its associated tracks.

The reconstruction and identification of electrons and muons is carried out using standard CMS algorithms, described in more detail in Refs. [36–40]. Reconstruction of the muon track starts from two tracks, one built in the silicon tracker and one built in the muon system. Hits used to reconstruct the tracks in the two systems are then used to reconstruct a track spanning over the entire detector [36]. Muon candidates are required to have a transverse momentum of ${p_{\mathrm {T}} > 45 ~\text {GeV}}$ with a measured uncertainty of ${\delta (p_{\mathrm {T}})/p_{\mathrm {T}} < 0.3}$ and must fall into the acceptance of the trigger of ${|\eta |<2.1}$. The candidate’s track must have transverse and longitudinal impact parameters with respect to the primary vertex position of less than 0.2 and 0.5$~\text {cm}$, respectively. At least one hit in the pixel detector, six or more hits in silicon-strip tracker layers, and matched segments in at least two muon detector planes are required to be associated with the reconstructed track. In order to suppress backgrounds from muons within jets, the scalar $p_{\mathrm {T}}$ sum of all other tracks within a cone of size 0.3 in ${\varDelta R=\sqrt{{(\varDelta \eta )^{2}+(\varDelta \phi )^{2}}}}$ (where $\phi$ is the azimuthal angle in radians) around the muon candidate’s track is required to be less than 10 % of the candidate’s $p_{\mathrm {T}}$.

In the electron reconstruction, ECAL clusters are matched to silicon pixel detector hits, which are then used as seeds for the reconstruction of tracks in the tracker. Electron candidates are built from clusters with associated tracks and must lie within the barrel or endcap acceptance regions, with pseudorapidities of $|\eta |<$ 1.442 and 1.56 $<|\eta |<$ 2.5, respectively, with a transverse energy $E_{\mathrm {T}} >35~\text {GeV}$. The transverse energy is defined as the magnitude of the projection on the plane perpendicular to the beam of the electron momentum vector normalized to the electron energy measured in the ECAL. Misidentification of jets as electrons is suppressed by requiring that the scalar sum of the $p_{\mathrm {T}}$ of all other tracks in a cone of size 0.3 in ${\varDelta R}$ around the electron candidate’s track is less than 5$~\text {GeV}$ . In addition, the sum of the $E_{\mathrm {T}}$ of calorimeter energy deposits in the same cone that are not associated with the electron candidate must be less than 3 % of the candidate’s $E_{\mathrm {T}}$ (plus a small $\eta$-dependent offset). To minimise the impact of additional $\mathrm {p}\mathrm {p}$ interactions in the same bunch crossing (pileup) on the selection efficiency, the calorimeter isolation is corrected for the average energy density in the event  [41]. Further reduction of electron misidentification is achieved by requiring the transverse profile of the energy deposition in the ECAL to be consistent with the expected electron profile, and the sum of HCAL energy deposits in a cone of size 0.15 in $\varDelta R$ to be less than 5 % of the electron’s ECAL energy. The transverse impact parameter of the electron candidate’s track with respect to the primary vertex must not exceed 0.02$\text {\,cm}$ and 0.05$\text {\,cm}$, for barrel and endcap candidates, respectively, and the track must not have more than one missing hit in the layers of the pixel detector it crossed.

The trigger efficiency has been measured using the “tag-and-probe” technique in dimuon events from Z decays described in  [36, 38, 39]. The trigger efficiency for muons that pass the selection requirements is ${92.9~\%}$ within ${|\eta | < 0.9}$, ${83.1~\%}$ within ${0.9< |\eta | < 1.2}$, and ${80.3~\%}$ within ${1.2< |\eta | < 2.1}$. The muon identification efficiency, including the isolation requirement, is measured with the tag-and-probe technique applied to muons from $\mathrm{Z}$ boson decays using tracks in the inner silicon tracker as probes. The same efficiency of ${95 \pm 1~\%}$ (syst) is obtained in the three pseudorapidity regions ${|\eta | < 0.9}$, ${0.9< |\eta | < 1.2}$, and ${1.2< |\eta | < 2.1}$, with corresponding efficiency ratios between data and the simulation of $0.990 \pm 0.005$ (syst), $0.992 \pm 0.005$ (syst), and $0.995 \pm 0.005$ (syst). A $p_{\mathrm {T}}$ range up to $300~\text {GeV}$ has been probed with the tag-and-probe method and the muon identification efficiencies remain constant within the statistical precision, as do the corresponding efficiency ratios between data and simulation. The evolution of the muon reconstruction and identification efficiencies and the muon trigger efficiency for muon $p_{\mathrm {T}} >300~\text {GeV}$ is based on simulation. Using dielectron events from $\mathrm{Z}$ boson decays [37], the total efficiency to reconstruct and select electrons with $p_{\mathrm {T}} ^{\mathrm {e}}>100~\text {GeV}$ is found to be ${88 \pm 2}$ % (syst) in the barrel region and ${84 \pm 4~\%}$ (syst) in the endcaps. According to Monte Carlo (MC) simulation, the variation of these efficiencies with electron $p_{\mathrm {T}}$ is less than ±1 % in the barrel and ±2 % in the endcaps. The corresponding efficiency ratios for $p_{\mathrm {T}} ^{\mathrm {e}}>100~\text {GeV}$ between data and simulation are $0.985 \pm 0.014$ (syst) in the barrel and $0.981 \pm 0.004$ (syst) in the endcaps. These efficiencies and efficiency ratios have been measured up to an electron $p_{\mathrm {T}}$ of 1$~\text {TeV}$ in the barrel and 500$~\text {GeV}$ in the endcap regions.

In the event selection, at least one isolated muon and one isolated electron that both pass the identification criteria described above are required. After the application of all efficiency scale factors that correct the simulation to the efficiencies measured in data, the combined dilepton reconstruction and identification efficiency for RPV $\tilde{\nu }_{\mathrm {\tau }}$ signal events within the detector acceptance is expected to be 80.6 % at ${M_{\tilde{\nu }_{\mathrm {\tau }}} =200~\text {GeV}}$ and the full selection efficiency including the trigger requirement is 71.2 %. The MC simulation predicts that this efficiency is constant within 3 % for masses between 200 $~\text {GeV}$ and 3 $~\text {TeV}$. The electron and the muon are not required to have opposite charge, in order to avoid a loss in signal efficiency due to possible electron charge misidentification at high electron $p_{\mathrm {T}}$. Since highly energetic muons can produce bremsstrahlung resulting in an associated supercluster in the calorimeter in the direction of the muon’s inner track, they can be misidentified as electrons. Therefore, an electron candidate is rejected if there is a muon with $p_{\mathrm {T}}$ greater than $5~\text {GeV}$ within $\varDelta R <0.1$ of the candidate. Only one $\mathrm {e}$ ${\mu }$ pair per event is considered. For about $1~\%$ of the events passing the event selection there is more than one $\mathrm {e}$ ${\mu }$ pair in the event, in which case the pair with the highest invariant mass is selected.

## Signal simulation

**Fig. 1:**
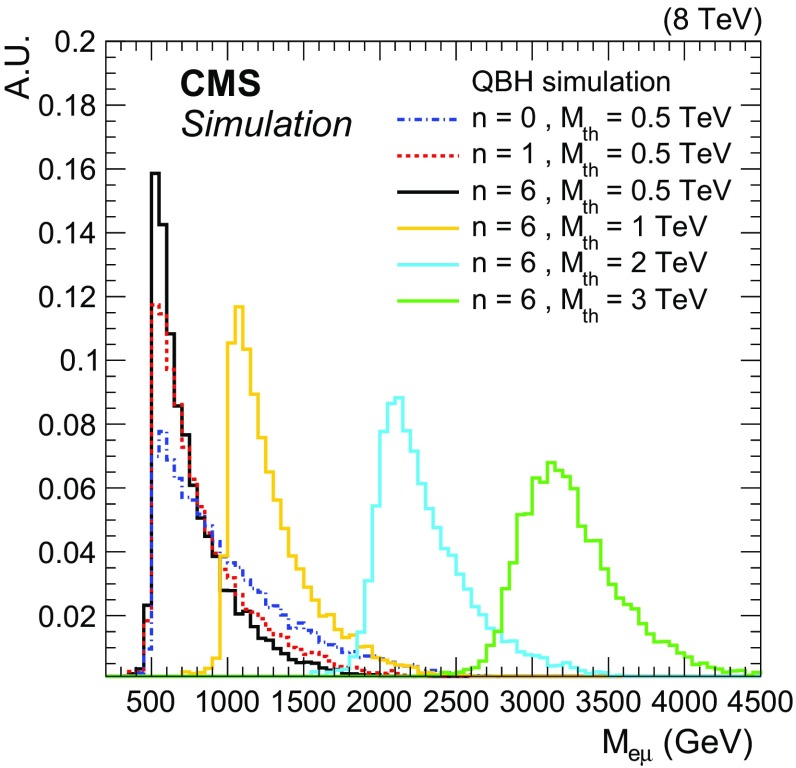
Invariant mass distributions of reconstructed $\mathrm {e}$ ${\mu }$ pairs from simulated QBH signal events that pass the event selection, normalized to unit area. The steps at the threshold masses ${M_{\mathrm {th}}}$ are smeared out by the detector resolution

The RPV and QBH signal samples are generated with the CalcHEP  (v. 3.4.1) event generator [42]. A cross section calculation at next-to-leading order (NLO) in perturbative QCD is used for the RPV signal [43], in which the factorization and renormalization scales are set to $M_{\tilde{\nu }_{\mathrm {\tau }}}$ and the CTEQ6M [44] set of parton distribution functions (PDF) is used. The invariant mass distributions of reconstructed $\mathrm {e}$ ${\mu }$ pairs from simulated QBH signal samples are presented in Fig. 1 for different signal masses and numbers of extra dimensions. A more detailed description of the implemented QBH model including the dependence of the $M_{\mathrm {e}{}{\mu }{}}$ spectrum from QBH decays on the model parameters is presented in Ref. [45]. The LFV ${\mathrm {Z}}^\prime$ signal events are produced with the MadGraph  (v. 5.1.5.9) generator [46]. The effects of the interference resulting from the $M_{{\mathrm {Z}}^\prime {}}=M_{\gamma {}^\prime {}}$ mass degeneracy on the cross section and signal acceptance are taken into account, and the coupling parameters of the model are taken to be the same as in Ref. [3]. All signal samples use the CTEQ6L1 [44] PDF, pythia  (v. 6.426) [47] for hadronization with the underlying event tune Z2*, and are processed through a simulation of the full CMS detector based on Geant4  (v. 9.4) [48]. The pythia Z2* tune is derived from the Z1 tune [49], which uses the CTEQ5L PDF set, whereas Z2* adopts CTEQ6L.

The total acceptance times efficiency for each of the three signal models considered in this analysis is determined using MC simulation with selection efficiencies corrected to the values measured in data. The signal acceptance, as defined by the selection on the lepton $p_{\mathrm {T}}$ and $\eta$ applied to the generated leptons in the signal simulation, and the product of acceptance and selection efficiency, are shown in Tables 1 and 2, evaluated for selected signal masses. The acceptance of the RPV $\tilde{\nu }_{\mathrm {\tau }}$ model is that of a generic spin-0 resonance. In the case of the LFV ${\mathrm {Z}}^\prime$ model, the acceptance is more model-specific due to the interference between the ${\mathrm {Z}}^\prime$ and the $\gamma {}^\prime$. This interference shapes the $\eta$ distributions of the leptons in the final state, which leads to a smaller acceptance compared to a generic spin-1 resonance. Table 3 lists the parameterizations of the acceptance times efficiency as a function of signal mass for the RPV $\tilde{\nu }_{\mathrm {\tau }}$ and LFV ${\mathrm {Z}}^\prime$ resonance signals, resulting from fits in the mass range from $200~\text {GeV}$ to $2.5~\text {TeV}$. These parameterizations are used later in the statistical interpretation of the resonance search.

**Table 1:** Signal acceptance (*A*) and the product of acceptance and efficiency ($A \epsilon$) for different signal masses, for the RPV $\tilde{\nu }_{\mathrm {\tau }}$ and LFV ${\mathrm {Z}}^\prime$ models. The acceptance is defined as the fraction of signal events in the simulation passing the selection on lepton $p_{\mathrm {T}}$ and $\eta$ applied to the generated leptons

$M_{\tilde{\nu }_{\mathrm {\tau }}}$ ($\text {TeV}$)	*A*	$A \epsilon$	$M_{{\mathrm {Z}}^\prime {}}$ ($\text {TeV}$)	*A*	$A \epsilon$
0.2	0.59	0.42	0.25	0.57	0.39
0.5	0.80	0.58	0.5	0.72	0.51
1.0	0.89	0.64	1.0	0.83	0.59
1.5	0.91	0.65	1.5	0.87	0.61
2.0	0.92	0.65	2.0	0.89	0.62

**Table 2:** Signal acceptance (*A*) and the product of acceptance and efficiency ($A \epsilon$) for different threshold masses $M_{\mathrm {th}}$, for the QBH models with $n=0$ and $n=6$ extra dimensions. The acceptance is defined as the fraction of signal events in the simulation passing the selection on lepton $p_{\mathrm {T}}$ and $\eta$ applied to the generated leptons

$n=0$			$n=6$		
$M_{\mathrm {th}}$ ($\text {TeV}$)	*A*	$A \epsilon$	$M_{\mathrm {th}}$ ($\text {TeV}$)	*A*	$A \epsilon$
0.5	0.85	0.61	0.5	0.82	0.60
1.0	0.90	0.63	1.0	0.89	0.64
2.0	0.93	0.64	2.0	0.93	0.65
3.0	0.94	0.63	3.0	0.94	0.64
4.0	0.94	0.62	4.0	0.94	0.63

**Table 3:** Parametrization of the product of signal acceptance and efficiency ($A \epsilon$) as a function of signal mass *M*, for the RPV $\tilde{\nu }_{\mathrm {\tau }}$ and LFV ${\mathrm {Z}}^\prime$ models. The value of *M* is expressed in units of $~\text {GeV}$

Model	Functional form of $A \epsilon$
RPV $\tilde{\nu }_{\mathrm {\tau }}$	$0.76 - 86.9/(61.4 + M) - 3.3 \times 10^{-5}~M$
LFV ${\mathrm {Z}}^\prime$	$0.74 - 141.3/(165.6 + M) - 2.7 \times 10^{-5}~M$

## Background estimation

The SM backgrounds contributing to the $\mathrm {e}$ ${\mu }$ final state can be divided into two classes of events. The first class comprises events with at least two prompt, isolated leptons. The second class consists of events with either jets or photons that are misidentified as isolated leptons, and events with jets containing non-prompt leptons. This second class of background is referred to as “non-prompt background” in this paper. The expected SM background from processes with two prompt leptons is obtained from MC simulations. It consists mostly of events from $\mathrm{t}\overline{\mathrm{t}}$ production and $\mathrm {W}$ $\mathrm {W}$ production; the former process is dominant at lower masses and the latter becomes equally important above $M_{\mathrm {e}{}{\mu }{}}\sim 1~\text {TeV}$. Other background processes estimated from MC simulation are the additional diboson processes $\mathrm {W}$ $\mathrm{Z}$ and $\mathrm{Z}$ $\mathrm{Z}$, single top $\mathrm{t}$ $\mathrm {W}$ production, and Drell–Yan (DY) $\mathrm {\tau }$ $\mathrm {\tau }$ events with subsequent decay of the $\mathrm {\tau }$ $\mathrm {\tau }$ pair into an electron and a muon. The $\mathrm{t}\overline{\mathrm{t}}$ , $\mathrm{t}$ $\mathrm {W}$, and $\mathrm {W}$ $\mathrm {W}$ simulated samples are generated using powheg  (v. 1.0) [50–52] with the CT10 PDF [53], and the DY, $\mathrm {W}$ $\mathrm{Z}$, and $\mathrm{Z}$ $\mathrm{Z}$ background samples are generated using the MadGraph  (v. 5.1.3.30) event generator with the CTEQ6L1 PDF. All background samples use pythia  (v. 6.426) for hadronization with the underlying event tune $\text{ Z2 }^{*}$. The generated events are processed through a full simulation of the CMS detector based on Geant4  (v. 9.4). Pileup interactions are included in the simulation and event-dependent weights are applied in order to reproduce the number of $\mathrm {p}\mathrm {p}$ interactions expected for the measured instantaneous luminosity. After this procedure, the distribution of the number of vertices per event observed in data is well described by the simulation. The simulated samples are normalized to the integrated luminosity of the data sample, $19.7~\text{ fb }^{-1}$. The cross sections are calculated to next-to-next-to-leading order (NNLO) accuracy in perturbative QCD for $\mathrm{t}\overline{\mathrm{t}}$ [54] and DY [55] and to NLO accuracy for the $\mathrm{t}$ $\mathrm {W}$ [56], $\mathrm {W}$ $\mathrm {W}$, $\mathrm {W}$ $\mathrm{Z}$, and $\mathrm{Z}$ $\mathrm{Z}$ [57] processes.

The main sources of non-prompt background in the $\mathrm {e}$ ${\mu }$ selection arise from $\mathrm {W}$+jet and $\mathrm {W}$ $\gamma$ production with a jet or photon that are misidentified as an electron. The $\mathrm{Z}$ +jet, QCD multijet, and $\mathrm{t}\overline{\mathrm{t}}$ processes yield subleading contributions to the background with non-prompt leptons. The $\mathrm {W}\gamma$ background is estimated from simulation based on the MadGraph  (v. 5.1.3.30) event generator. A background estimation based on control samples in data, using the jet-to-electron misidentification rate (MR) method explained below, is used to determine the $M_{\mathrm {e}{}{\mu }{}}$ distributions from $\mathrm {W}$+jet and QCD multijet production. The measurement of the jet-to-electron misidentification rate has been carried out in the context of Ref. [40]. It starts from a sample collected using a prescaled single electromagnetic cluster trigger, in which the presence of an electron candidate with relaxed electron identification criteria is required. The events of the sample must have no more than one reconstructed electron with ${E_{\mathrm {T}} > 10~\text {GeV}}$, in order to suppress the contribution from $\mathrm{Z}$ decays. The misidentification measurement can be biased by selecting genuine electrons from $\mathrm {W}$+jet events or converted photons from $\mathrm {\gamma }$+jet events. Processes that can give a single electron, such as $\mathrm{t}\overline{\mathrm{t}}$, $\mathrm{t}$ $\mathrm {W}$, $\mathrm {W}$ $\mathrm {W}$, $\mathrm {W}$ $\mathrm{Z}$, $\mathrm{Z} \rightarrow \mathrm {\tau }{}\mathrm {\tau }{}$, and $\mathrm{Z} \rightarrow \mathrm {e}\mathrm {e}$  where, if a second electron is produced, it fails to be reconstructed, give another less significant source of contamination. Simulated samples are used to correct for this contamination and its effect on the MR. After these corrections, the electron MR, measured in bins of $E_{\mathrm {T}}$ and $\eta$, is the number of electrons passing the full selection over the number of electron candidates in the sample.

**Fig. 2:**
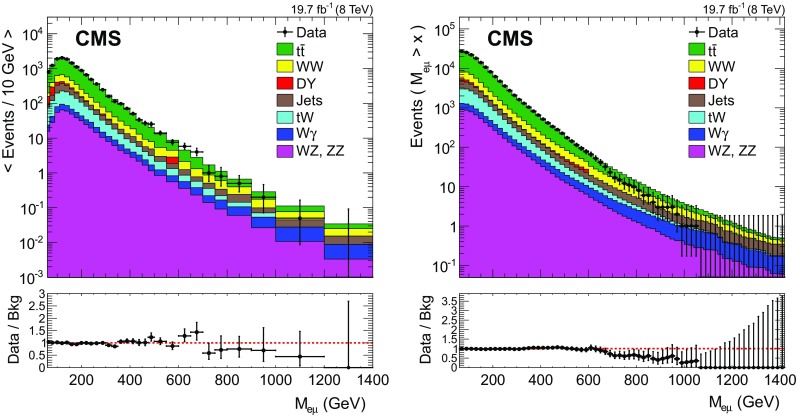
The invariant mass distribution of selected $\mathrm {e}$ ${\mu }$ pairs (*left*), and the corresponding cumulative distribution, where all events above the mass value on the *x*-axis are summed (*right*). The *points with error bars* represent the data and the *stacked histograms* represent the expectations from SM processes. The label ‘Jets’ refers to the estimate of the $\mathrm {W}$+jet and QCD multijet backgrounds from data. The ratio of the data to the background for each bin is shown at the *bottom*. The *horizontal lines* on the data points indicate the bin width

**Table 4:** The number of observed events compared to the background expectation in five invariant mass ranges and in the full invariant mass range. The yields obtained from simulations are normalized according to their expected cross sections. The background label ‘Jets’ refers to the estimate of the $\mathrm {W}$+jet and QCD multijet backgrounds from data

	Total	Invariant mass ranges in units of $~\text {GeV}$				
		${<}200$	200–400	400–600	600–1000	${>}1000$
$\mathrm{t}\overline{\mathrm{t}}$	$20100 \pm 1800\phantom {0}$	$15800 \pm 1400\phantom {0}$	$4050\pm 450\phantom {0}$	$260 \pm 44\phantom {0}$	$30 \pm 7\phantom {0}$	$0.9 \pm 0.4$
$\mathrm {W}$ $\mathrm {W}$	$3150 \pm 260\phantom {0}$	$2400 \pm 200\phantom {0}$	$670 \pm 64\phantom {0}$	$68 \pm 8\phantom {0}$	$13 \pm 2\phantom {0}$	$0.9 \pm 0.2$
$\mathrm{t}$ $\mathrm {W}$	$2000 \pm 160\phantom {0}$	$1550 \pm 120\phantom {0}$	$430 \pm 40\phantom {0}$	$30 \pm 3\phantom {0}$	$4 \pm 0.5$	${<}0.2$
$\text{ Jets }$	$1570 \pm 470\phantom {0}$	$1250 \pm 400\phantom {0}$	$280 \pm 83\phantom {0}$	$30 \pm 9\phantom {0}$	$5 \pm 2$	$0.6 \pm 0.3$
DY	$960 \pm 100$	$910 \pm 100$	$40 \pm 15$	$5 \pm 5$	${<}1$	${<}0.1$
$\mathrm {W}$ $\mathrm{Z}$/$\mathrm{Z}$ $\mathrm{Z}$	$940 \pm 80\phantom {0}$	$670 \pm 60\phantom {0}$	$240 \pm 20\phantom {0}$	$27 \pm 3\phantom {0}$	$5 \pm 0.6$	$0.3 \pm 0.1$
$\mathrm {W}$ $\gamma$	$480 \pm 240$	$360 \pm 180$	$100 \pm 50\phantom {0}$	$12 \pm 6\phantom {0}$	$3 \pm 1.5$	$0.6 \pm 0.3$
Total bkg	$29200 \pm 2300\phantom {0}$	$22900 \pm 1800\phantom {0}$	$5800 \pm 560\phantom {0}$	$430 \pm 53\phantom {0}$	$60 \pm 9\phantom {0}$	$3.5 \pm 0.6$
Data	28925	22736	5675	448	65	1

Using the measured electron MR, the $\mathrm {W}$+jet and QCD multijet contributions can be estimated from a sample with a muon passing the single-muon trigger and the full muon selection, and an electron candidate satisfying the relaxed selection requirements but failing the full electron selection. Each event in the sample is weighted by the factor $\mathrm {MR}/(1-\mathrm {MR})$ to determine the overall contribution of the jet backgrounds. Contributions from processes other than $\mathrm {W}$+jet and QCD multijet are subtracted from the sample to which the MR is applied, to avoid double counting. This subtraction is based on MC simulated background samples. A systematic uncertainty of 30 % is applied to the jet background estimate, based on cross-checks and closure tests. An uncertainty of 50 % is assigned to the background estimate for the $\mathrm {W}$ $\gamma$ process, which is taken from simulation at leading order (LO) in perturbative QCD.

## Results

After the event selection, 28 925 events are observed in data. The $\mathrm {e}$ ${\mu }$ invariant mass distribution is shown in Fig. 2, together with the corresponding cumulative distribution. A comparison of the observed and expected event yields is given in Table 4. The dominant background process is $\mathrm{t}\overline{\mathrm{t}}$ , which contributes 69 % of the total background yield after selection, followed by $\mathrm {W}$ $\mathrm {W}$ production, contributing 11 %. The two selected leptons carry opposite measured electric charge in 26 840 events and carry the same charge in 2085 events. According to the background estimation, $2100\pm 360$ events with same-charge $\mathrm {e}$ ${\mu }$ pairs are expected, most of which stem from the $\mathrm {W}$+jet process, followed by $\mathrm{t}\overline{\mathrm{t}}$ and diboson production $\mathrm {W}$ $\mathrm{Z}$/$\mathrm{Z}$ $\mathrm{Z}$.

The systematic uncertainties assigned to backgrounds obtained from simulation include the integrated luminosity (2.6 %) [58] and the acceptance times efficiency (5 %). The latter is based on the uncertainties in the various efficiency scale factors that correct the simulation to the efficiencies measured in data. According to simulation, the evolution of the lepton selection efficiencies from the $\mathrm{Z}$ pole, where they are measured, to high lepton $p_{\mathrm {T}}$ is covered within this uncertainty. The uncertainty in the muon momentum scale is 5 % per $~\text {TeV}$ . Electron energy scale uncertainties are 0.6 % in the barrel and 1.5 % in the endcap. These momentum and energy scale uncertainties cumulatively lead to an uncertainty in the total background yield of 2 % at $M_{\mathrm {e}{}{\mu }{}}=500~\text {GeV}$ and 3.5 % at $M_{\mathrm {e}{}{\mu }{}}=1~\text {TeV}$. Uncertainties in the electron $E_{\mathrm {T}}$ and muon $p_{\mathrm {T}}$ resolutions have a negligible impact on the total background yield. The uncertainty associated with the choice of PDF in the background simulation is evaluated according to the PDF4LHC prescription [59, 60] and translates into an uncertainty in the background yield ranging from 5 % at $M_{\mathrm {e}{}{\mu }{}}=200~\text {GeV}$ to 9 % at $M_{\mathrm {e}{}{\mu }{}}=1 ~\text {TeV}$. Among the uncertainties in the cross sections used for the normalization of the various simulated background samples, the 5 % uncertainty in the NNLO QCD cross section of the dominant $\mathrm{t}\overline{\mathrm{t}}$ background [54] is the most relevant. Further uncertainties associated with the modelling of the shape of the $\mathrm {e}{}{\mu }{}$ invariant mass distribution are taken into account for the two leading backgrounds: $\mathrm{t}\overline{\mathrm{t}}$ (higher-order corrections on the top-$p_{\mathrm {T}}$ description discussed in [61]) and $\mathrm {W}$ $\mathrm {W}$ (scale uncertainties studied with the powheg generator). These lead to an uncertainty in the total background yield of up to 13 % at $M_{\mathrm {e}{}{\mu }{}}=1~\text {TeV}$. A further systematic uncertainty arises from the limited sizes of the simulated background samples at high invariant mass, where the background expectation is small. Taking all systematic uncertainties into account, the resulting uncertainty in the background yield ranges from 9 % at $M_{\mathrm {e}{}{\mu }{}}=200~\text {GeV}$ to 18 % at $M_{\mathrm {e}{}{\mu }{}}=1~\text {TeV}$.

As shown in the cumulative invariant mass distribution in Fig. 2, we observe a deficit in data compared to the background expectation for $M_{\mathrm {e}{}{\mu }{}}\ge 700~\text {GeV}$. In this invariant mass region, 17 events are observed and the background estimate yields $27\pm 4$ (syst) events. Combining the systematic and statistical uncertainties, the local significance of this discrepancy is below 2$\sigma$.

No significant excess with respect to the expectation is found in the measured $\mathrm {e}$ ${\mu }$ invariant mass distribution, and we set limits on the product of signal cross section and branching fraction for signal mass hypotheses above $200~\text {GeV}$. Two types of signal shapes are considered for the limit setting: a narrow resonance and the broader $\mathrm {e}$ ${\mu }$ invariant mass spectrum from QBH decays. The RPV $\tilde{\nu }_{\mathrm {\tau }}$ and ${\mathrm {Z}}^\prime$ signals both result in a narrow resonance. For coupling values not excluded by existing searches, the intrinsic widths of these signals are small compared to the detector resolution. Therefore, Gaussian functions are used to model the signal shapes. For each probed resonance signal mass, the two parameters, acceptance times efficiency (Table 3) and invariant mass resolution, define the signal shape used for limit setting. The invariant mass resolution is derived from fits of Gaussian distributions to the $\mathrm {e}$ ${\mu }$ invariant mass spectra from MC simulated signal samples and ranges from 1.6 % at a resonance mass of ${M_{\text {res}}=200~\text {GeV}}$ to 6 % at ${M_{\text {res}}=3~\text {TeV}}$. For high values of $\mathrm {e}$ ${\mu }$ pair invariant mass, it is dominated by the resolution on the measurement of the muon $p_{\mathrm {T}}$, which ranges from about 2 % at ${p_{\mathrm {T}} =200~\text {GeV}}$ to 6 % at ${p_{\mathrm {T}} =500~\text {GeV}}$ and 10 % at ${p_{\mathrm {T}} =1~\text {TeV}}$. These values are obtained from MC simulations and agree within the uncertainties with measurements using cosmic ray muons. This model of the narrow resonance allows for a scan of the invariant mass spectrum with a fine spacing of the signal mass hypothesis that corresponds to the invariant mass resolution.

Unlike the $\tilde{\nu }_{\mathrm {\tau }}$ and ${\mathrm {Z}}^\prime$ signals, the QBH signal exhibits a broader shape with a sharp edge at the threshold mass $M_{\mathrm {th}}$ and a tail towards higher masses (Fig. 1). The QBH signal shapes are obtained directly from simulated samples.

The systematic uncertainties in the signal entering the limit calculation are the 2.6 % uncertainty in the integrated luminosity, the 5 % uncertainty in the product of acceptance and efficiency, and the relative uncertainty in the mass resolution, which ranges from 2 % at ${M_{\text {res}}=200~\text {GeV}}$ to 40 % at ${M_{\text {res}}=3~\text {TeV}}$. The uncertainty in the signal acceptance times efficiency is dominated by the uncertainty in the trigger, lepton reconstruction, and identification efficiencies, and includes the subleading PDF uncertainty in the signal acceptance.

Upper limits at $95~\%$ CL on the product of cross section and branching fraction are determined using a binned likelihood Bayesian approach with a positive, uniform prior for the signal cross section [62]. The signal and background shapes enter the likelihood with a binning of 1$~\text {GeV}$, well below the invariant mass resolution for masses above 200 GeV. For the resonant signals $\tilde{\nu }_{\mathrm {\tau }}$ and ${\mathrm {Z}}^\prime$ , search regions in the invariant mass spectrum are defined as ${\pm } 6$ times the invariant mass resolution evaluated at the hypothetical resonance mass. Only events in these search regions enter the binned likelihood in the limit calculation. The impact of a further broadening of the signal window size on the median expected limit has been found to be negligible within the uncertainties. For mass hypotheses above 800$~\text {GeV}$, the upper bound of the search region is dropped. In the case of the QBH signal, the search region is defined by a lower bound at ${M_{\mathrm {th}}-6\sigma _M}$, where $\sigma _M$ is the invariant mass resolution, and there is no upper bound. The nuisance parameters associated with the systematic uncertainties are modelled with log-normal distributions, and a Markov Chain MC method is used for integration. For each mass hypothesis considered, the posterior probability density function is derived as a function of the signal cross section times branching fraction and yields the 95 % CL upper limit on this parameter of interest.

**Fig. 3:**
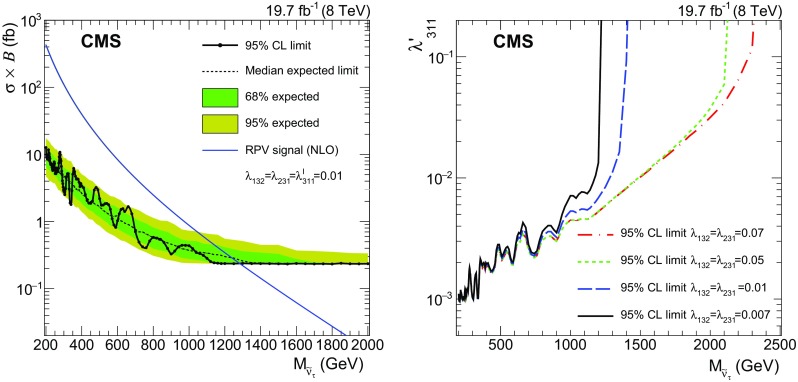
*Left* The $95~\%$ CL upper limit on the product of signal cross section and branching fraction for the RPV $\tilde{\nu }_{\mathrm {\tau }}$ signal as a function of the mass of the resonance $M_{\tilde{\nu }_{\mathrm {\tau }}}$. *Right* The 95 % CL limit contours for the RPV $\tilde{\nu }_{\mathrm {\tau }}$ signal in the ($M_{\tilde{\nu }_{\mathrm {\tau }}}$, $\lambda '_{311}$) parameter plane. The values of the parameter $\lambda _{132} =\lambda _{231}$ are fixed to 0.07 (*red dashed and dotted*), 0.05 (*green small-dashed*), 0.01 (*blue dashed*), and 0.007 (*black solid*). The regions above the *curves* are excluded

The $95~\%$ CL limits on the signal cross section times branching fraction for the RPV $\tilde{\nu }_{\mathrm {\tau }}$ resonance signal are shown in Fig. 3 (left). The signal cross section shown is calculated at NLO in perturbative QCD with the RPV couplings set to $\lambda _{132} =\lambda _{231} =0.01$ and $\lambda '_{311} =0.01$. For these couplings, a lower mass limit of 1.28$~\text {TeV}$ is obtained. At this mass, the observed limit on the cross section times branching fraction is 0.25$\text {\,fb}$. For a comparison with earlier searches at hadron colliders [20, 22], the two coupling benchmarks $\lambda _{132} =\lambda _{231} =0.07$, $\lambda '_{311} =0.11$ and $\lambda _{132} =\lambda _{231} =0.05$, $\lambda '_{311} =0.10$ are considered. For RPV couplings $\lambda _{132} =\lambda _{231} =0.07$ and $\lambda '_{311} =0.11$, we set a mass limit of 2.30$~\text {TeV}$, and improve the lower bound of 2.0$~\text {TeV}$ previously set [22]. The lower bound on the signal mass for $\lambda _{132} =\lambda _{231} =0.05$ and $\lambda '_{311} =0.10$ is 2.16$~\text {TeV}$. In the narrow width approximation, the cross section times branching fraction scales with the RPV couplings as:

$$\begin{aligned} \sigma \mathcal {B}&\sim \left( \lambda '_{311} \right) ^2[\left( \lambda _{132} \right) ^2+\left( \lambda _{231} \right) ^2]/(3\left( \lambda '_{311} \right) ^2\\&\quad +[\left( \lambda _{132} \right) ^2+\left( \lambda _{231} \right) ^2]). \end{aligned}$$

**Fig. 4:**
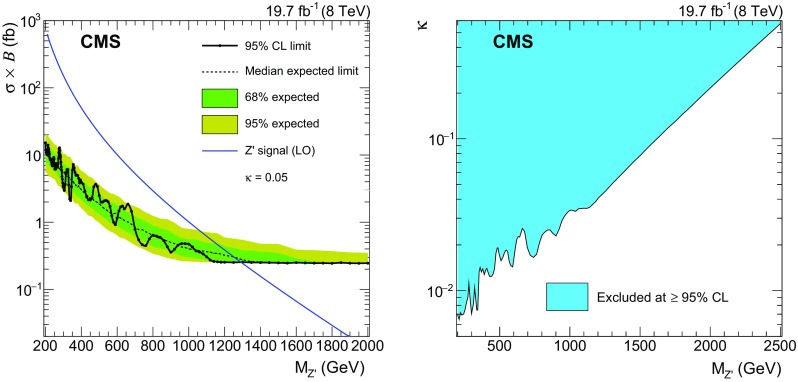
*Left* The $95~\%$ CL exclusion limit on the product of signal cross section and branching fraction for the ${\mathrm {Z}}^\prime$ signal as a function of the mass $M_{{\mathrm {Z}}^\prime }$. *Right* The 95 % CL limit contour for the ${\mathrm {Z}}^\prime$ signal in the ($M_{{\mathrm {Z}}^\prime }$, $\kappa$) parameter plane

Using this relation and the observed upper cross section bounds, we derive the limit contour in the ${(M_{\tilde{\nu }_{\mathrm {\tau }}},~\lambda '_{311})}$ parameter plane as a function of a fixed value of $\lambda _{132} =\lambda _{231}$. For the results presented in Fig. 3 (right), values of the couplings $\lambda '_{311}$ and ${\lambda _{132} =\lambda _{231}}$ up to 0.2 and 0.07 are considered, respectively. The ratio of decay width to mass of the $\mathrm {\tau }$ sneutrino is less than 0.5 % for these coupling values and finite-width effects are small. Searches for resonant dijet production [27, 29] that cover the $\mathrm {\tau }$ sneutrino decay to a $\mathrm{d}$ $\overline{\mathrm{d}}$ pair via the coupling $\lambda '_{311}$ do not exclude this region of parameter space. In the model considered here with resonant production of the $\tilde{\nu }_{\mathrm {\tau }}$, we do not reach the sensitivity of muon conversion experiments, which lead to a bound on the coupling product of ${\lambda _{132} \lambda '_{311} <3.3 \times 10^{-7} (M_{\tilde{\nu }_{\mathrm {\tau }}}/1~\text {TeV})^{2}}$ at 90 % CL, assuming ${\lambda _{132} =\lambda _{231}}$. For comparison, with a signal mass of ${M_{\tilde{\nu }_{\mathrm {\tau }}} =1~\text {TeV}}$ and the assumption ${\lambda _{132} =\lambda _{231} =\lambda '_{311}}$, we obtain a limit of ${\lambda _{132} \lambda '_{311} <4.1 \times 10^{-5}}$ at 90 % CL. We present results in terms of the product of the production cross section and branching fraction of the $\tilde{\nu }_{\mathrm {\tau }}$ that do not depend on a specific production mechanism of the sneutrino.

The $95~\%$ CL limits on the signal cross section times branching fraction for the ${\mathrm {Z}}^\prime$ signal, which exhibits a different acceptance from the spin-0 resonance in the RPV model, are presented in Fig. 4 (left). For the coupling modifier $\kappa =0.05$, a lower bound on the signal mass ${M_{{\mathrm {Z}}^\prime {}}=M_{\gamma {}^\prime {}}}$ of 1.29$~\text {TeV}$ is obtained. Figure 4 (right) shows the corresponding limit contour in the ${(M_{{\mathrm {Z}}^\prime },~\kappa )}$ parameter plane. Since this resonance is produced dominantly in the ${\mathrm{d}\overline{\mathrm{s}}}$ initial state, the bound from searches for muon conversion is not as strong as for the RPV $\tilde{\nu }_{\mathrm {\tau }}$ signal, but searches for ${\text{ K }^{0}_{\mathrm {L}} \rightarrow \mathrm {e}{}{\mu }{}}$ decays yield a stringent exclusion limit of ${\kappa \lesssim M_{{\mathrm {Z}}^\prime {}}/100~\text {TeV}}$ at 90 % CL. This can be compared to our bound of ${\kappa =0.031}$ at 90 % CL for ${M_{{\mathrm {Z}}^\prime {}}=M_{\gamma {}^\prime }=1~\text {TeV}}$.

**Fig. 5:**
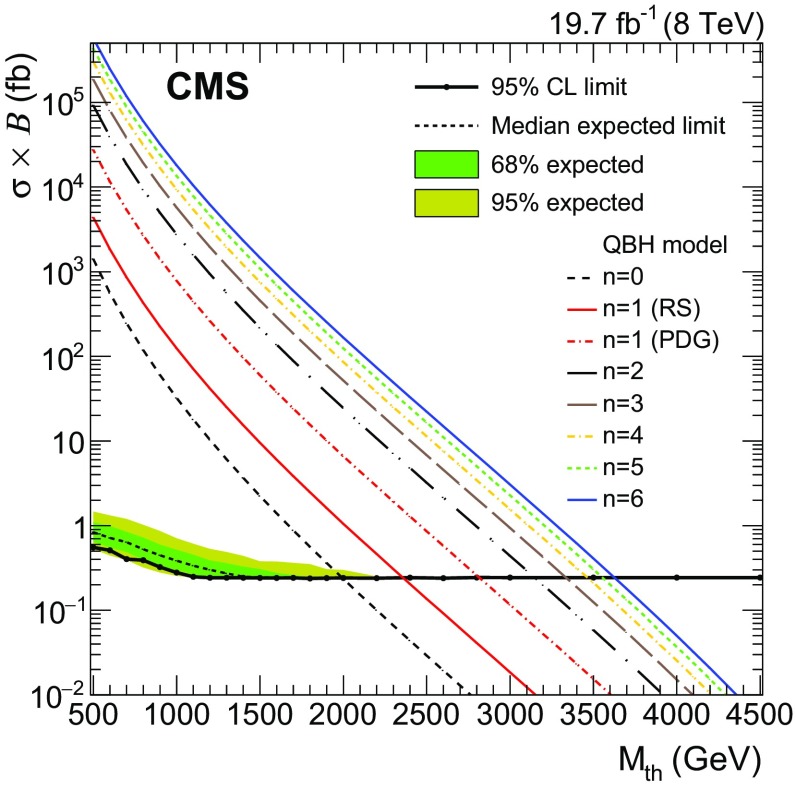
The $95~\%$ CL exclusion limit on the product of signal cross section and branching fraction for the QBH signal as a function of the threshold mass $M_{\mathrm {th}}$. The limits have been calculated using the signal shape of the QBH model without extra dimensions ($n=0$). For signal masses ${M_{\mathrm {th}}\ge 1~\text {TeV}}$, the change in the QBH signal shape for different numbers of extra dimensions has a negligible impact on the limit

In the QBH search, we set limits on the mass threshold for QBH production, $M_{\mathrm {th}}$, in models with $n=0$ to $n=6$ extra dimensions. The $95~\%$ CL limits on the signal cross section times branching fraction for the QBH signal are shown in Fig. 5. For $n=0$ in a model with a Planck scale at the TeV scale from a renormalization of the gravitational constant, we exclude QBH production below a threshold mass $M_{\mathrm {th}}$ of 1.99$~\text {TeV}$. For $n=1$, two signal cross sections are considered with the Schwarzschild radius evaluated in the RS and PDG conventions. The resulting limits on $M_{\mathrm {th}}$ are 2.36$~\text {TeV}$ and 2.81$~\text {TeV}$, respectively. For ADD-type black holes with $n>1$, we obtain lower bounds on $M_{\mathrm {th}}$ ranging from 3.15$~\text {TeV}$ for $n=2$ to 3.63$~\text {TeV}$ for $n=6$. A summary of the 95 % CL lower mass limits set for all signal models is presented in Table 5.

**Table 5:** The ${95~\%}$ CL observed and expected lower bounds on the signal masses of $\mathrm {\tau }$ sneutrinos in RPV SUSY, resonances in the LFV ${\mathrm {Z}}^\prime$ model, and QBHs, each with subsequent decay into an $\mathrm {e}$ ${\mu }$ pair. For the QBH signal with $n=1$, two signal cross sections are considered with the Schwarzschild radius evaluated in either the Randall–Sundrum (RS) or the Particle Data Group (PDG) convention

Signal model	Lower limit signal mass (TeV)	
	Observed	Expected
RPV $\tilde{\nu }_{\mathrm {\tau }}$ ($\lambda _{132} = \lambda _{231} = \lambda '_{311} =0.01$)	1.28	1.24
RPV $\tilde{\nu }_{\mathrm {\tau }}$ ($\lambda _{132} = \lambda _{231} = 0.05,~\lambda '_{311} =0.10$)	2.16	2.16
RPV $\tilde{\nu }_{\mathrm {\tau }}$ ($\lambda _{132} = \lambda _{231} = 0.07,~\lambda '_{311} =0.11$)	2.30	2.30
LFV ${\mathrm {Z}}^\prime$ ($\kappa =0.05$)	1.29	1.25
QBH $n=0$	1.99	1.99
QBH $n=1$ (RS)	2.36	2.36
QBH $n=1$ (PDG)	2.81	2.81
QBH $n=2$	3.15	3.15
QBH $n=3$	3.34	3.34
QBH $n=4$	3.46	3.46
QBH $n=5$	3.55	3.55
QBH $n=6$	3.63	3.63

## Summary

A search has been reported for heavy states decaying promptly into an electron and a muon using 19.7$~\text {fb}^\text {-1}$ of proton-proton collision data recorded with the CMS detector at the LHC at a centre-of-mass energy of $8~\text {TeV}$. Agreement is observed between the data and the standard model expectation with new limits set on resonant production of $\mathrm {\tau }$ sneutrinos in R-parity violating supersymmetry with subsequent decay into $\mathrm {e}$ ${\mu }$ pairs. For couplings ${\lambda _{132} =\lambda _{231} =0.01}$ and ${\lambda '_{311} =0.01}$, $\mathrm {\tau }$ sneutrino lightest supersymmetric particles for masses $M_{\tilde{\nu }_{\mathrm {\tau }}}$ below ${1.28~\text {TeV}}$ are excluded at 95 % CL. For couplings ${\lambda _{132} =\lambda _{231} =0.07}$ and ${\lambda '_{311} = 0.11}$, masses $M_{\tilde{\nu }_{\mathrm {\tau }}}$ below ${2.30~\text {TeV}}$ are excluded. These are the most stringent limits from direct searches at high-energy colliders. For the ${\mathrm {Z}}^\prime$ signal model, a lower mass limit of $M_{{\mathrm {Z}}^\prime }=M_{\gamma {}^\prime }={1.29~\text {TeV}}$ is set at 95 % CL for the coupling modifier $\kappa =0.05$. This direct search for resonant production of an $\mathrm {e}$ ${\mu }$ pair at the TeV scale does not reach the sensitivity of dedicated low-energy experiments, but complements such indirect searches and can readily be interpreted in terms of different signals of new physics involving a heavy state that decays promptly into an electron and a muon. Lower bounds are set on the mass threshold for the production of quantum black holes with subsequent decay into an $\mathrm {e}$ ${\mu }$ pair in models with zero to six extra dimensions, assuming the threshold mass to be at the Planck scale, ranging from ${M_{\mathrm {th}}=1.99~\text {TeV} ~(n=0)}$ to ${3.63~\text {TeV} ~(n=6)}$. These are the first limits on quantum black holes decaying into $\mathrm {e}$ ${\mu }$ final states.
